# CareKnowDo—A Multichannel Digital and Telephone Support Program for People With Chronic Kidney Disease: Feasibility Randomized Controlled Trial

**DOI:** 10.2196/33147

**Published:** 2023-11-23

**Authors:** Riley Elizabeth Reston, Fergus J Caskey, Barnaby Hole, Udaya Udayaraj, John Weinman

**Affiliations:** 1 Atlantis Health London United Kingdom; 2 King's College London London United Kingdom; 3 University of Bristol Bristol United Kingdom; 4 North Bristol National Health Service Trust Bristol United Kingdom; 5 Oxford University Hospitals National Health Service Foundation Trust Oxford United Kingdom; 6 Nuffield Department of Medicine University of Oxford Oxford United Kingdom; 7 School of Cancer & Pharmaceutical Sciences King's College London London United Kingdom

**Keywords:** kidney disease, chronic, blood pressure, randomized controlled trial, telemedicine, mobile health, mHealth, self-management, guideline adherence, medication adherence, illness beliefs, medication beliefs, health psychology, preventative medicine, qualitative research

## Abstract

**Background:**

Chronic kidney disease (CKD) is a common, progressive condition. Lifestyle changes and antihypertensive medication can slow the progression to end-stage kidney disease, which requires renal replacement therapy. However, adherence to these recommendations is often low.

**Objective:**

The aim of CareKnowDo was to assess the feasibility of rolling out a digital self-management support and adherence program integrated with a patient-facing electronic health record, Patient View (PV).

**Methods:**

A 2-arm, parallel, individual-level pragmatic feasibility pilot randomized controlled trial was conducted at 2 National Health Service (NHS) sites in the United Kingdom. A total of 61 patients with CKD were randomized 1:1 into 2 groups and provided with either a new, tailored digital and telephone support program (CareKnowDo: 31/61, 51%) integrated with PV or standard care (PV alone: 30/61, 49%). Quantitative measures included clinical and psychosocial measures. The primary outcomes were feasibility based: recruitment rate, dropout, and the exploration of associations.

**Results:**

Of the 1392 patients screened in local kidney clinics, 269 (19.32%) met the basic inclusion criteria; the first 22.7% (61/269) who met the eligibility criteria were recruited to participate in the study. Of the 69 patients, 23 (38%) patients completed the final 6-month follow-up web-based survey. Reasons for the attrition were explored. A higher belief in the ability of the treatment to control CKD was associated with lower blood pressure at baseline (*r*=0.52; *P*=.005), and a higher perceived understanding of CKD at baseline was associated with lower blood pressure at follow-up (*r*=0.66; *P*<.001). Beliefs about medicines at baseline were associated with blood pressure at baseline but not at follow-up. This was true for both concerns about medicines (*r*=0.58; *P*=.001) and perceived necessity of medicines (*r*=0.42; *P*=.03).

**Conclusions:**

A tailored digital and nurse call–based program to enhance support for patients with CKD was piloted in 2 NHS sites and found to be feasible and acceptable. However, to maximize the effectiveness of the intervention (and of future trials), consideration should be given to the target audience most likely to benefit, as well as how to help them access the program as quickly and easily as possible.

**Trial Registration:**

NHS Health Research Authority, IRAS ID 184206; https://www.hra.nhs.uk/planning-and-improving -research/application-summaries/research-summaries/careknowdo-pilot-version-1/

## Introduction

### Clinical Context

Chronic kidney disease (CKD) is a complex, long-term condition that in 2017 was estimated to have a global prevalence of 9.1% [1]. This figure is on the rise, and with increase in the proportion of the aging population, it is set to reach 16.7% by 2030 [2]. It occurs when the kidney function is impaired and is generally progressive, with later stages being associated with higher rates of cardiovascular disease, anemia, and metabolic bone disease.

Once the disease has progressed to end-stage kidney disease (ESKD), the condition is severely life-limiting. Symptoms of ESKD, and side effects of its treatment, include a high prevalence of depression, fatigue, pain, muscle cramps, difficulty sleeping, and sexual dysfunction [3]. The 5-year survival rate is 74.5% for people aged <65 years and 32.5% for people aged ≥65 years. The relative risk of death compared with the general population was 21 for patients aged 35 to 39 years and 1.5 for those aged >85 years [4].

The early stages of CKD are generally asymptomatic and unproblematic for the individual’s daily life. As the condition progresses, it produces more noticeable symptoms and impairs quality of life [5]. Progression is generally continuous over the lifespan, and controlling blood pressure forms a core part of treatment recommendations [6,7]. In the United Kingdom, the largest burden on the health care system comes when patients reach ESKD; transplants cost approximately UK £12,000 (US $14,973) per patient per year (across the estimated lifetime of the patient), and hemodialysis costs around UK £27,000 (US $ 33,686) per patient per year [8]. This represents over half the estimated cost to the National Health Service (NHS) for CKD care, whereas patients with ESKD represent 1 in 50 patients with CKD.

Helping patients to avoid or delay progression through the stages of CKD, especially ESKD, therefore, represents not only a huge prospective quality of life boost but also large health care savings. Because CKD also causes increasing impairment of quality of life as it progresses, controlling blood pressure is a key aspect of care for people with CKD, preventing further morbidity and mortality [9,10]. People with CKD are at an increased risk for cardiac events, and good blood pressure control can reduce this risk in people with and without CKD [11].

### Adherence to Prescribed Treatment

Despite the importance of medication in slowing the progression of CKD, adherence to these treatments is often low [12].

For example, across common chronic conditions globally, it has been estimated that between 4% and 31% of patients never fill their first prescription and only 50% to 70% of people take their medications regularly (at least 80% of the time) [13]. This appears to be particularly true of asymptomatic conditions, in which the perceived need for treatment is low [14]. For example, in hypertension, only 25% to 64% of people were estimated to be adherent to their prescribed statin treatment [13].

Nonadherence to prescribed treatment matters. For example, in hypercholesterolemia (a common risk factor for CKD), medication nonadherence was associated with a 25% increased risk of hospitalization and a further 25% increased risk of mortality after hospital discharge [15].

This can result in ineffective treatments and increase health care costs [12]. Increased hospitalization and urgent care to address preventable disease progression increase health care costs. Diabetes is the common comorbidity in ESKD. In the United States, people living with diabetes who had low levels of adherence had annual health care costs nearly double that of those with higher adherence [13]. Adherence has been associated with cost savings across common chronic conditions [16]. Hypercholesterolemia and diabetes are both common risk factors for CKD and its comorbidities.

Although pharmacological treatment is a key element of treatment for hypertension, adherence to broader recommended self-management behaviors also plays a role. In the United Kingdom, the National Institute for Health and Care Excellence guidelines recommend that all patients with CKD be provided with information about their CKD diagnosis; the opportunity for shared decision-making; and self-management education of blood pressure, smoking cessation, exercise, and diet, in addition to relevant medicines [7].

### Interventions to Improve Treatment Adherence

Interventions to improve self-management behavior and adherence to treatment are becoming increasingly common and can be delivered through many channels in countries with high levels of technological use [17]. Although face-to-face and phone call–based interventions can be effective [18], they can be costly and difficult to scale up to large populations, such as those with CKD. Remote interventions with some automated components delivered using the web, email, and SMS can provide a more scalable alternative but sometimes lack the human element that some patients find helpful [19,20]. A potential solution is to tailor the intervention received by each patient so that those with greater need receive more support, via different channels, than those with lower need [17,21,22].

Evidence shows that patients make decisions about their treatment in line with their beliefs about their illness and treatment rather than the objective state of their condition [23,24]. The Common-Sense Model of Self-Regulation has been developed over several decades of research and can be used to help develop and evaluate health behavior change interventions [25]. Such interventions aim to modify patient behavior by first modifying patient beliefs.

### This Study

Helping patients to be more involved in their own care (self-management) is publicly claimed to be the core principle of the United Kingdom’s NHS, although widespread implementation of these ideals has been patchy [26]. One service helping to involve patients in the United Kingdom is a service called *Patient View* (PV), which has been in operation since 2004 [27]. PV is run by the Renal Association.

PV allows people being treated for kidney disease by the NHS to view the results of their clinical tests, such as blood and urine tests, as well as recent letters from their nephrologist. Part of the rationale for this is to support people living with CKD to make decisions, and to take actions, that will reduce the likelihood of progression to ESKD. However, PV provides little information about what to do with this information or how to translate it into behavior change, such as reducing salt intake, adhering to medicines, or increasing physical activity.

CareKnowDo is a multichannel support program that aims to address this gap. It was developed by a team of doctoral-level psychologists, specializing in health behavior at Atlantis Health, in collaboration with the Renal Association. CareKnowDo aims to provide people living with CKD with tailored web, SMS, email, and nurse phone support based on key self-management behaviors. It also features integration with PV so that the results can be viewed directly on the CKD site.

As CareKnowDo (with PV integration) was a novel intervention, the core aim of this study was to establish the feasibility of rolling this out in NHS kidney units and of assessing its effectiveness in a fully powered randomized controlled trial (RCT). The feasibility of trial methods was assessed through patient flow (including number of eligible patients, uptake, number willing to be randomized, and number of assessments completed). Differences between the control and intervention groups over time were explored to provide estimates for powering future work in this area. Implementation facilitators and barriers were explored qualitatively, along with measures of intervention engagement, to further inform future intervention implementation and trial design [28]. The trial design and reporting were guided by the Pilot and Feasibility extension of the 2010 CONSORT (Consolidated Standards of Reporting Trials) guidelines [29].

## Methods

### Trial Design

This study was a 2-arm, parallel, individual-level pragmatic feasibility pilot RCT of CareKnowDo plus PV versus PV alone. It was conducted at 2 NHS sites in the United Kingdom (NHS Health Research Authority, IRAS ID 184206) [30].

### Proposed Solution and Hypotheses

To attempt to address the problem of low adherence to lifestyle and medication recommendations among people with CKD, we developed a multichannel support program (web, email, SMS, and phone). The solution was integrated with PV to allow participants to access their clinical test results via the CareKnowDo website.

To test the intervention, a 2-arm RCT was established to test CareKnowDo (a complex intervention) [31] plus PV (intervention arm) versus PV alone (control arm).

### Participants

Inclusion and exclusion criteria for the study are listed in Textbox 1.

Initially, participants in each arm were to be stratified into *prevalent* and *incident*, with a recruitment target of 30 for each group (to capture potential differences between these groups). It was purported that the intervention would be particularly valuable for newer patients. Patients were considered *prevalent* if they had been invited to attend 3 nephology outpatient clinics in the last 12 months and attended nephrology outpatient clinics at least twice previously.

However, owing to a lack of patients being recruited in the incident stratum, this stratification requirement was dropped, and the final sample consisted predominantly of patients who were prevalent (54/61, 88%).

Patients received an invitation to participate in the study 2 weeks before their next scheduled clinic appointment. The research nurse (RN) followed this up with a phone call 1 week before their clinic appointment to check whether they were interested and to answer any questions. Recruitment took place face to face at the clinic during the patient’s scheduled appointment, during which the baseline information was collected. The patient information sheet included information on the process of randomization into one of the 2 groups and a broad idea of what the supportive intervention and control groups would entail. Neither group was given in-depth details about the contents of CareKnowDo until after randomization.

**Eligibility criteria.**

Inclusion criteria

- The patients must
  - have a diagnosis of chronic kidney disease
    - estimated glomerular filtration rate of 15 to 59 on the last measurement, or latest urine albumin creatinine ration/urine protein creatinine ratio of >29 mg/mmol
  - be aged at least 18 years
  - be able to read and speak English (as the pilot intervention was only in English)
  - be computer literate, for example, have their own email address that they use themselves
  - have access to the internet and mobile phone
  - currently, be treated with antihypertensive medication

Exclusion criteria

- The patients must
  - be deemed by their clinician to be likely to need kidney replacement therapy (such as dialysis) within the next 6 months
  - have severe or profound intellectual impairments and learning difficulties

### Randomization

The participants were randomized 1:1 to the intervention group or the control group. A randomization list was generated in R statistical software (v3.3.1; The R Foundation) [32] using the package randomizeR (v1.4) [33], which was locked and digitally signed before study commencement. A separate randomization list was produced for each of the 2 study sites. A block randomization approach was used, with random-sized blocks of 2 to 4. After patients had consented to participate in the study, the RN sent the patient ID to the study coordinator, who allocated the patients sequentially per randomization list.

### Follow-Up

The initial plan for follow-up was 12 months, but because recruitment took longer than anticipated, this was adjusted to 6 months.

### Interventions

#### Overview

Owing to PV integration, patients in both arms needed to have a PV log-in before they could proceed. Once randomized, patients in both the control and intervention arms were sent an email inviting them to sign up to PV on the web. The email had link that led them to a survey, used for both baseline measurement and program personalization. Upon completion of the questionnaire, they were taken to the PV home page (PV group) or to the home page for CareKnowDo (CareKnowDo group).

Materials were appropriate for a literacy level between ages 11 and 14.

#### PV Website

PV is a website that gives patients direct access to their latest test results and other information such as physicians’ letters. It is available to most UK patients with renal disorders, but patients must sign up through their renal service.

PV allows patients to remotely log into a secure website that can relay their latest clinical information such as blood test results and physicians’ letters. Helping patients to be more involved in their care by making them more aware of their results may improve outcomes [34]. However, uptake is often low, and the site does not offer any behavioral support beyond the results themselves. In addition, it is important to support patients with resources and education to help them interpret the results that they receive via patient portals such as PV. A study found that 65% of patients incorrectly interpreted the risk presented by their results as presented in a hypothetical scenario and would likely have taken an inappropriate action (calling their physician immediately, making an appointment within the next 4 weeks, or waiting 3 months for their next appointment) [35].

#### CareKnowDo

The patients in the CareKnowDo arm had access to a website with 3 distinct modules. These were:

1. Mind Matters: designed to address low mood;
2. Lifestyle Matters: addressing primarily diet, exercise, and how these affect CKD; and
3. Medication Matters: covering adherence to antihypertensive medication.

These modules were selected as 3 areas in which self-management behaviors can affect hypertension, as well as each other. Each module included internet-based activities and tools based on cognitive behavioral therapy and other evidence-based behavior change techniques. They also contained psychoeducational content designed to educate patients about CKD and address key unhelpful beliefs that impact self-management behavior. The patients were also provided with an inbound nurse line for queries or concerns.

The approach to developing the intervention, such as behavior change techniques selected and methods of translating them into interventional content, was guided by the Behavior Change Wheel and Behavior Change Technique taxonomy from the Centre for Behaviour Change [1-3]. The order in which the patients were directed to these 3 modules was determined by how they answered the questionnaire at baseline. These were supported by SMS and emails on each of these topics, directing the patient to the site that supported these topics.

Patients who scored lower on Necessity and higher on Concerns about medication, based on their scores on the Beliefs about Medicines Questionnaire (BMQ; see *Data Collection and Outcomes* below), were allocated to a “High-risk” profile and would receive additional nurse calls. Patients could opt out of any of the individual channels, or the program altogether. Each patient was enrolled in the program for 6 months, and after that time point email, SMS, calls, and tailoring of the website stopped.

A reminder email was sent to patients who did not engage with the intervention within the last month.

Multimedia Appendix 1 shows example screens from the website.

### Data Collection and Outcomes

Demographic details including age, sex, ethnicity, and age of leaving full-time education were collected via the CareKnowDo website. A wide range of clinical details was collected, including CKD stage, number of prescribed medications, comorbidities, estimated glomerular filtration rate (a measure of kidney function), and glycated hemoglobin A_1c_ (HbA_1c_). In addition, the following psychological measures were captured through web-based self-assessment:

1. *BMQ* [36]: based on the Necessity-Concerns Framework of treatment beliefs, this gives an indication of how necessary a person thinks their medicine is (5 items) and how concerned they are about it (5 items);
2. *Brief Illness Perceptions Questionnaire (B-IPQ)* [37]: based on the Common-Sense Model of Illness Representations, this measures patients’ beliefs about specific aspects of their illness (eg, identity, personal control, treatment control, consequences, emotional impact, timeline, and coherence) using single-item scales (score range: 1-10); and
3. *Patient Health Questionnaire-9 (PHQ-9)* [38]: a screening measure of depressive symptoms used in both research and clinical practice in the United Kingdom.

The full list of measures can be found in Multimedia Appendix 2 [36-41].

### Outcomes

As this is a feasibility study, no single primary end point was selected. The data used to assess feasibility included the following: uptake, that is, what proportion of patients chose to participate and reasons for declining; willingness for patients to be randomized; response rates to follow-up questionnaires; number of patients enrolled per month; and means and SDs for the outcome measures (eg, blood pressure) to allow the estimation of the sample size for a full-powered RCT.

Qualitative interviews were conducted with 5 participants after they had completed the 6-month trial period. Interviews were conducted by RER by phone and lasted for approximately 30-40 minutes. A semistructured interview guide was followed, and thematic analysis was applied to the findings.

### Sample Size

The sample size was determined pragmatically to be a suitable number to assess feasibility.

### Analysis Approach

Owing to the feasibility nature of the study, the statistical analysis was predominantly descriptive. Indications of efficacy were investigated using correlation and regression conducted in R statistical software. Exploratory correlational analysis controlled for multiple comparisons using Bonferroni correction. An intention-to-treat approach was used when dropout occurred, and missing data were not imputed.

Qualitative interviews were directly audio coded using the principles of thematic analysis [42].

### Ethics Approval and Informed Consent

Approval for the study was given by the London Dulwich Research Ethics Committee on December 14, 2015 (reference 15/LO/1700). Written consent was required for all patients before their participation in the study. The participants were free to withdraw from the study at any time.

## Results

### Participant Flow

**Figure 1:**
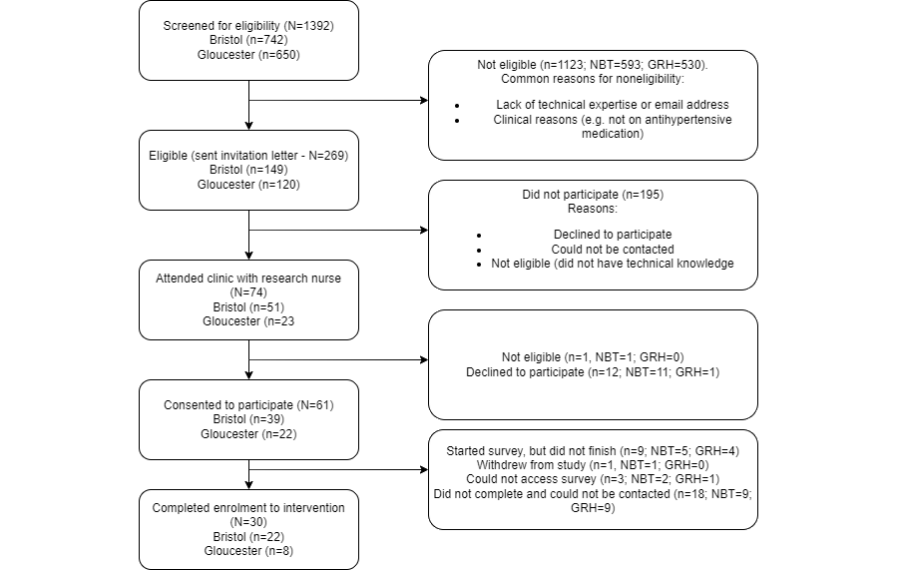
Participant flow from screening to randomization. GRH: Gloucestershire Royal Hospital; NBT: North Bristol National Health Service Trust.

Figure 1 shows the participant flow from the initial screening of patient records to enrollment in the intervention by site. A high proportion of the patients (1123/1392, 80.68%) were ineligible to participate in the study. Qualitative feedback from the RNs attributed this to a range of factors with respect to patients, including low computer literacy and not currently being prescribed an antihypertensive medication, primarily because of not being on antihypertensive medications. Of the 269 patients identified as potentially eligible from the initial record screening, the first 74 (27.5%) potential participants who had met the eligibility criteria and had agreed to the study in principle over the phone met with the RN in clinic and completed the informed consent process, until the recruitment target (61/74, 82%) was met.

**Figure 2:**
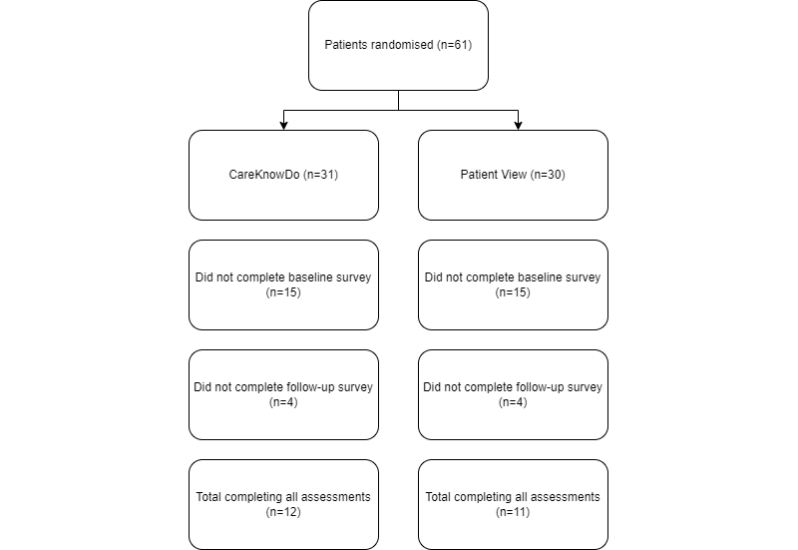
Patient flow after randomization.

Figure 2 shows the patient flow after randomization. In each arm, roughly half of the participants did not complete the enrollment into the intervention after being randomized. Follow-up RN calls revealed that the most common reasons were as follows: not receiving a PV log-in before attempting to enroll; losing invitation emails in spam folders; not checking emails; and technical difficulties while logging in.

Of the patients who completed baseline measurements, most of them also completed follow-up measurements 6 months later (intervention arm: 12/31, 39%; control arm: 11/30, 37%).

### Demographic Characteristics

Table 1 shows the demographic characteristics of each intervention group at baseline and follow-up. There were no significant differences between the groups at baseline.

**Table 1:** Demographic characteristics by intervention group at baseline.

			CareKnowDo (n=31)		Patient View (n=30)
Sex (male), n (%)			20 (65)		22 (73)
Age (years), mean (SD)			56.5 (15.7)		61.0 (15.6)
Systolic blood pressure (mm Hg), mean (SD)			138.4 (17.0)		134.6 (15.4)
eGFR^a^ (mL/min/1.73 m^2^), mean (SD)			34.2 (15.8)		32.4 (13.1)
**Length of time on blood pressure medication (count), n (%)**					
	>2 years	10 (32)		9 (30)	
	1-2 years	5 (16)		1 (3)	
	6-12 months	0 (0)		1 (3)	
	1-3 months	0 (0)		1 (3)	
	Unsure	0 (0)		3 (10)	

^a^eGFR: estimated glomerular filtration rate.

### Outcomes

#### Uptake

Patient flow from screening to enrollment is shown in Figure 1. Out of patients with CKD who were screened, a total of 80.67% (1123/1392) were eligible to participate. Qualitative feedback from the RNs conducting the screening attributed this to a range of factors with respect to patients, including low computer literacy or availability or not currently being prescribed antihypertensive medication. Of the remaining 269 patients who were sent an invitation letter, 74 (27.5%) agreed for the RN to attend their next kidney clinic to discuss regarding participating in the study in person.

#### Willingness for Patients to Be Randomized

No objections to being randomized between intervention groups were noted for this study.

#### Number of Patients Enrolled per Month

It took site 1 a total of 5 months to recruit 39 patients (average of 7.8 per month) and site 2 a total of 9 months to recruit 22 patients (average of 2.4 per month).

Once recruited into the study, of the 61 patients, only 31 (51%) patients completed the web-based baseline questionnaire, which was circulated via email. Thus, although these patients were enrolled in the study, they did not complete the intervention.

#### Reasons for Low Uptake

RNs at each recruiting site captured the following reasons for low incident patient recruitment: (1) there were fewer incident patients coming through their clinics than prevalent patients; (2) incident patients frequently did not meet the inclusion criteria. For example, they were often not on antihypertensive medications; and (3) some incident patients, or their carers, did not fully acknowledge that they had a chronic kidney condition.

#### Response Rates to Follow-Up Questionnaires

Once patients were fully enrolled and had completed the baseline questionnaire, of the 61 patients who started the study, 23 (38%) patients completed the follow-up questionnaire at 6 months. This constituted 74% (23/31) of the patients who completed the intervention.

Because of the high rate of noncompletion of the baseline survey (30/61, 49%), the clinical differences between the 2 groups were analyzed. No significant differences in baseline clinical variables were detected between those who completed the baseline questionnaire and those who did not (highest Pearson *r*=0.19, not significant for HbA_1c_; combined logistic regression model of blood pressure, estimated glomerular filtration rate, HbA_1c_, sex, and age did not significantly predict baseline survey completion).

#### Telephone Engagement

Attempts were made to reach patients via phone if they did not complete the baseline survey after a reminder email had been sent. Many patients could not be contacted by phone after 3 attempts. Follow-up revealed that 1 potential cause could be patients being at work at the time that calls were attempted (9 AM-5 PM on weekdays), as many patients included in the study were of working age.

#### Estimating Outcome Parameters

Table 2 shows clinical and psychological variables at baseline and follow-up. Systolic blood pressure did not significantly change from baseline to follow-up in either the intervention or the control group when analyzing patients who completed baseline enrollment to the intervention (*P*=.68; *df*=25). The mean blood pressure in both arms was <140 mm Hg per arm, which is within the target range recommended by the United Kingdom’s National Institute for Health and Care Excellence [43], the United States Kidney Disease: Improving Global Outcomes, and the Kidney Disease Outcomes Quality Initiative guidelines.

**Table 2:** Clinical and psychological variables by group at baseline and follow-up.

			Baseline, mean (SD)		Follow-up, mean (SD)	
			CareknowDo	Patient View	CareKnowDo	Patient View
Systolic blood pressure (mm Hg)			139.6 (19.0)	133.5 (11.0)	137.2 (14.9)	135.7 (18.9)
eGFR^a^ (mL/min/1.73 m^2^)			34.0 (14.7)	33.0 (12.6)	34.4 (17.1)	31.8 (13.8)
HbA_1c_^b^			47.3 (16.9)	56.9 (24.5)	46.2 (15.9)	58.3 (26.0)
**Psychosocial**						
	**Illness perceptions**					
		Personal control	4.5 (2.5)	2.4 (2.3)	4.2 (2.1)	4.2 (2.6)
		Treatment control	6.8 (2.7)	7.9 (2.4)	5.3 (2.1)	7.2 (2.4)
		Illness concerns	5.4 (3.4)	6.3 (3.5)	6.3 (2.8)	6.4 (3.2)
		Illness consequences	3.4 (2.3)	3.2 (3.6)	4.1 (2.8)	3.8 (3.0)
		Illness understanding	6.1 (2.7)	6.4 (2.7)	6.3 (3.1)	6.5 (2.3)
	Beliefs about medicines (Concerns)		2.8 (0.8)	2.4 (1.1)	2.8 (0.8)	2.7 (0.8)
	Beliefs about medicines (Necessity)		3.5 (0.8)	3.8 (0.9)	3.6 (0.6)	3.7 (1.1)
	Self-efficacy		7.4 (2.2)	7.1 (2.2)	6.7 (2.2)	5.8 (2.5)
	Depressive symptoms		2.5 (0.6)	2.6 (0.5)	3.0 (0.5)	2.9 (0.5)

^a^eGFR: estimated glomerular filtration rate.

^b^HbA_1c_: glycated hemoglobin A_1c_.

#### Exploratory Outcomes Analysis

Part of the rationale of the program was the use of illness perceptions and beliefs about medicines to tailor the program, with a view to reducing blood pressure at follow-up. Table 3 shows correlations between blood pressure, illness perceptions, and treatment beliefs.

Perceptions of how much treatment can improve one’s condition were negatively correlated with systolic blood pressure at baseline; people who believed that treatment could help had lower baseline blood pressure (Pearson *r*=−0.52; *P*=.005), but this relationship was not present at follow-up. Conversely, perceived understanding of illness at baseline was negatively correlated with systolic blood pressure at follow-up (Pearson *r*=−0.66; *P*<.001). Someone who feels that they understand their illness better has a lower blood pressure 6 months later. The relationship between Understanding and Blood pressure was not present at baseline (*r*=−0.31; *P*=.12).

Beliefs about medicines at baseline were associated with blood pressure at baseline, but not at follow-up. This was true for both concerns about medicines (*r*=0.58; *P*=.001) and perceived necessity of medicines (*r*=0.42; *P*=.03). Patients who had higher concerns about medicines, and those who perceived medication to be more necessary, had higher blood pressure at baseline.

**Table 3:** Pearson correlations between blood pressure (BP) and psychological variables with *P* values presented in parentheses.

	BP baseline	BP follow-up	Personal control	Illness affect	Treatment control	Concern about illness	Illness understanding	BMQ^a^ concerns
BP baseline	—^b^	—	—	—	—	—	—	—
BP follow-up	0.46 (.001)	—	—	—	—	—	—	—
Personal control	−0.04 (.83)	−0.01 (.94)	—	—	—	—	—	—
Illness affect	0.14 (.50)	−0.26 (.19)	0.24 (.23)	—	—	—	—	—
Treatment control	−0.52 (.005)	−0.08 (.71)	0.26 (.18)	0.05 (.79)	—	—	—	—
Concern about illness	0.06 (.76)	−0.13 (.54)	0.12 (.55)	0.72 (< .001)	0.16 (.43)	—	—	—
Illness understanding	−0.31 (.12)	−0.66 (< .001)	0.21 (.30)	0.46 (.02)	0.39 (.046)	0.24 (.24)	—	—
BMQ Concerns	0.58 (.001)	−0.11 (.61)	−0.02 (.92)	0.27 (.18)	−0.42 (.03)	0.08 (.67)	−0.13 (.52)	—
BMQ Necessity	0.42 (.03)	0.22 (.29)	−0.11 (.59)	0.33 (.09)	−0.03 (.90)	0.51 (.007)	−0.03 (.87)	0.28 (.16)

^a^BMQ: Beliefs about Medicines Questionnaire.

^b^Not applicable.

### Qualitative Feedback

Qualitative feedback was obtained in the form of free text comments in the 6-month follow-up survey available to all participants and 5 in-depth qualitative interviews with patients from the intervention arm.

Generally, patients leaving comments found the service helpful but pointed out some areas for improvement (Table 4).

Some of the core feedback obtained outside of the formal qualitative research was that enrolling in the program was difficult because of the requirement for a PV log-in.

Consistent with this, a theme of the interviews was the perception that the program would be more useful for people in 2 situations: those early on who had just been diagnosed and those with more advanced CKD. In other words, the least useful scenario would be for someone with early-stage CKD but has been aware of this diagnosis for some time—the group of patients most prevalent in this study. This is evident in the following excerpt from the interview:

And how many times would you say you went on the website?

Investigator

About three or four times... and that would have been towards the early part of the study, where I was still a bit unsure about what was going on with my condition.

P1

One transplant patient had been under the care of the same nephrologist for 23 years and noted that at this point, they did not need help in managing their condition. They had attended kidney care seminars early on and seemed highly knowledgeable about their condition and engaged in their care. Regarding the program, they said the following:

Yes, I would have thought the survey... the program... I could have taken advantage of it had it been available to me when I was going through initial kidney failure and end stage renal failure.

P2

Another patient noted that they had also received extensive support with their condition, including renal counseling, but that the additional support was useful:

It was a little supplement to the huge amount of information I had from the nephrology department, but it was useful to have it there as a reassurance.

P3

When asked whether any of the SMS or email prompts were helpful, 1 participant had the following to say:

Yes one in particular... I was just going to go to a nephrology appointment and I think it said about communication etc, so I think that was helpful

P3

They said they did not feel that they needed support so much anymore, as they had been reassured about their prognosis. They found the ability to look up their blood tests results extremely helpful. The topic of the interview and discussion about the purpose of the program being preventative in nature prompted the respondent to say that they felt they should be more engaged in their care again.

Another patient said that the program was not relevant as they felt well:

Because of the position I’m in with my condition at the moment, I’m not that interested in the results... when, if, if and when, it’s not even an if, when it does start getting worse, then maybe I’ll start looking... but because it’s only every 6 months, and I go to the hospital and it’s either yes or no, you need to do this or whatever, I’m taking the advice of the hospital at the moment.

P1

However, later in the interview, they said that they were struggling to manage their weight, with lifestyle elements like this being part of what the program sets out to tackle. They did not make the connection between their broader lifestyle and CKD, seeing the treatment of their illness as separate from their self-management behaviors.

Yeah, my lifestyle doesn’t really... the diet is really difficult at the moment, because I work strange hours, I’m up at 4 o’clock in the morning, or working through till 4 o’clock in the morning, [...] I try to eat healthily, but when it’s 11 o’clock at night and you’re hungry and the only thing left open is your fast food joints, or your packaged sandwiches. It becomes very difficult to eat healthy.

These tell the story of support being most valuable to people early in the journey, but those people being the hardest to reach and engage with, and even if they can be reached, like P1, they might be struggling with an aspect of self-management but do not see the link between this and their CKD.

**Table 4:** Qualitative feedback.

Theme			Example comment
**Finding the service helpful**			
	Integration with PV^a^	“Very useful to have blood test results instantly” “Helps you see your results quickly”	
	Additional information, education, and lifestyle tips provided by CareKnowDo	“We’re living in an information age now. It’s about giving information back to the patients relatively easily, to help them understand exactly what’s going on with their condition” “It told me a bit more information that the doctor hadn’t mentioned about what I’ve got. It was fairly clear, and clear cut. All the information was pretty clearly displayed on there. And it was relatively easy to get to everything”	
	Providing a feeling of being supported	“(The program) enables you to monitor your condition via showing blood test result and providing information. This program provides reassurance that interest in you and your condition is ongoing and gives hope that your welfare is being considered”	
**Suggestions for improvement**			
	Readability and clarity of information	“The nursing personnel that I have met are excellent. I find the website as scary as reading the information sheets that come with the medication. The website information seems to be written by medical experts and can only be understood by medical experts. Have you considered a review of the presentation by a panel of sufferers to see if they can understand the content?”	
	Perceived usefulness for asymptomatic condition	“I do not see the program being of great benefit to transplant patients” “Other people may find it more useful than I personally do”	
	Mode of delivery	“Better to speak to a person” “I haven’t felt well enough to stare a computer screen as it brings on the fatigue”	

^a^PV: Patient View.

## Discussion

### Summary

This feasibility study found that a remote intervention to support self-management of CKD was feasible to implement and was perceived as helpful by participants. The trial methods used were also found to be feasible, with the potential to be scaled up to an RCT of intervention efficacy. In line with the study aims, several areas for improvement were identified.

### Illness Identity and Coherence in Patients With Newly Diagnosed CKD

The low number of eligible and accepting patients who are considered *incident* is important to consider in the design of future interventions that attempt to target patients with CKD early. This needs to be further explored before conducting a larger trial. The fact that many patients in this category were not aware of their diagnosis despite having attended their specialist kidney clinic is a perplexing finding. That they may not be fully aware of their diagnosis demonstrates an important point about health literacy and disease education and raises important issues about how to best communicate with these patients to ensure better disease awareness and self-management.

It may be that the patients had not internalized their diagnoses. In terms of illness perceptions, these patients may not yet have a good sense of *illness identity* or *illness coherence*. This is something that future interventions and iterations of CareKnowDo can address. This is particularly important, as it is concordant with the qualitative findings from this study that some patients said that they did not feel that they needed the intervention, as their condition was not currently affecting them. The fact that the program was designed as preventative, aiming to slow the decline to ESKD, either did not sufficiently come across or was easily forgotten. This is in line with the finding that many people have a very poor understanding of what the kidneys do and how to protect them [44].

In asymptomatic conditions, nonadherence to treatment is particularly common [13], and this may extend to low engagement with services attempting to address the issue. Tackling low engagement is key to gaining traction for interventions hoping to make a difference in public health issues.

The difficulty of recruiting patients in this setting is not uncommon and can also occur in drug trials. A trial of sertraline for ESKD screened 709 patients; 63 initially screened for depression and underwent diagnostic interviews; 30 were identified as having depression; and only 21 then went on to complete the trial [45]. The realities of patient flow should always be considered when designing intervention trials, using conservative rather than optimistic predictions. Enhancing patient interest in the intervention and fostering an understanding of the importance of the condition should be established at the very beginning, when patients reach the intervention in the first place.

The content of the program was aimed at both prevalent and incident types of patients. It may be that the content was not advanced enough for people who had been living with the condition for a longer period. A future iteration of the program should tailor to whether patients are incident or prevalent or to perceived knowledge about the condition. In addition, if the program addresses patients not on antihypertensive medication, this should also be included in the tailoring process.

It appears that support from a service such as CareKnowDo was perceived as being most useful to newly diagnosed patients who had the most questions and had not had years to build up their own experiences of how to live with the disease. However, these are the patients who were most difficult to reach in this study partly because of a lack of acceptance of their condition. It may be that patients who perceive their current need to be low overestimate how well controlled their CKD is, as ongoing clinic attendance may reflect a high risk of progression. This may reflect unhelpful thoughts or behaviors.

### Treatment Necessity and Control

It is surprising that the B-IPQ question about Treatment Control was negatively correlated with blood pressure at baseline, whereas the BMQ Necessity subscale was positively correlated with it. B-IPQ Treatment Control and BMQ Necessity were not correlated with each other (*r*=−0.03; *P*=.90).

It may be that in this sample, the 2 measures tap into 2 slightly different underlying concepts. The B-IPQ item asks “How much do you think your treatment can help your illness?” whereas the BMQ necessity subscale asks about a range of issues to patients, such as whether life would be impossible without their medicine and whether their present health depends on their medicine. In the case of blood pressure control for CKD, in which consequences of nonadherence are likely to be years away, it is perhaps less surprising that “necessity” and “can help control” may elicit different patterns of responses. Furthermore, “treatment” may have been interpreted as holistic recommendations from the health care team, including lifestyle changes such as diet and exercise, rather than medicine alone.

### Conversion Rate of Participants Recruited in Clinic to Completing Web-Based Survey

The fact that only half of the patients recruited in the clinic completed the subsequent enrollment steps to use the intervention (if they were in the CareKnowDo arm) indicates that this process needs streamlining. Several factors could be addressed, including the following:

1. The enrollment questionnaire was 104 questions long.
2. The fact that some patients did not have a PV log-in before trying to log on to the site meant that many had tried to log in, but they could not and had to await their PV password before trying again. In several cases, this appears to have been the blocking factor.

Informal follow-up via the clinic revealed that the delay between being signed up to the study in the clinic and receiving a PV log-in (required to log in to the baseline survey) caused many patients to disengage from the service after failed attempts to log on.

It was noted by the RNs that participants could be difficult to reach by phone, suggesting that this may be due to the calls being made during working hours. Site clinicians noted that this may be unrepresentative, as at least 50% of the clinical population is older or retired.

High rates of attrition are common in internet interventions [46], with even landmark trials such as the trial by Etter (2005), who found that follow-up surveys were responded to by as few as 35% of participants [47]. To ensure that patients receive an effective “dose” of digital behavior change interventions, it is essential to promote maximum engagement as much as possible [48].

### Implications for Future Research and Practice

This study did not manage to create sufficient patient engagement at the start of the program. Gaining this early engagement means addressing perceptions of illness coherence from the introduction of the service to the patient and even earlier. Such programs designed to prevent future harm, rather than address an immediate set of symptoms or concerns, must be introduced in a way that conveys their purpose, which aligns with the patients’ perceptions of what their illness is, either by adjusting how the program is presented or by ensuring that the patient receives appropriate illness education, including fostering a stronger belief in the need for treatment at this relatively early stage. Streamlining the enrollment process for web-based programs is key, even in the context of research, where ongoing contact may increase motivation to persist when usability is suboptimal. Ensuring that when a patient signs up for support does not require a further waiting period to first log on is key, otherwise, engagement falls off quickly. When possible, patients should be directed to the service by a person at the point when they have everything they need to proceed.

In future, it may be that an intervention should be available to all people diagnosed with CKD, regardless of the use of antihypertensive medication, and should be able to “switch on” medication-specific components when they become relevant.

### Conclusions

A web-based support intervention aimed at promoting self-management and adherence to blood pressure medication was successfully rolled out in the UK health care setting. It was found to be feasible and acceptable, and the content was liked by participants. The pathway through which patients come to the intervention and the features that promote engagement should be key areas for development.

## Appendix

Multimedia Appendix 1 Measures collected at each time point.

Multimedia Appendix 2 Screens from the CareKnowDo website.

Multimedia Appendix 3 CONSORT eHEALTH checklist (V 1.6.1)

## Abbreviations

B-IPQ: Brief Illness Perceptions Questionnaire
BMQ: Beliefs about Medicines Questionnaire
CKD: chronic kidney disease
CONSORT: Consolidated Standards of Reporting Trials
ESKD: end-stage kidney disease
HbA_1c_: glycated hemoglobin A_1c_
NHS: National Health Service
PHQ-9: Patient Health Questionnaire-9
PV: Patient View
RCT: randomized controlled trial
RN: research nurse
