# Supplementary material for: CareKnowDo—A Multichannel Digital and Telephone Support Program for People With Chronic Kidney Disease: Feasibility Randomized Controlled Trial
Source: JMIR Form Res. 2023 Nov 23;7:e33147. doi: 10.2196/33147 (PMC10704307; doi:10.2196/33147)
Supplement: Multimedia Appendix 1 [file formative_v7i1e33147_app1.docx]

### Multimedia Appendix 1

| **Construct / measure** | **Baseline** | **6 months** |
| --- | --- | --- |
| Demographic details: Age, gender, ethnicity, age left full time education | X |  |
| Clinical details (self-report):   - CKD stage, Time diagnosed (CKD and BP) - Total number of prescribed meds - Prescribed BP medication(s): product, dose and regimen - Length of time on BP treatment - Length of time in secondary care - Co-morbidities (e.g. diabetes, CVD) - Unscheduled HCP visits | X | X |
| - Beliefs about medicines (Beliefs about Medicines Questionnaire)**[36]** - Illness perceptions (Brief Illness Perceptions Questionnaire)**[37]** - Mood (PHQ-9)**[38]** - Lifestyle activities (Diet, exercise and smoking habit question adapted from the diabetes self-care activities questionnaire) - Health related Quality of Life (POS-5 Renal Disease Scale & EQ5D) - Quality of communication with doctor (three items adapted from Medical Interview Satisfaction Scale, MISS-21) | X | X |
| Patient satisfaction with program |  | X |
