# Supplementary material for: CareKnowDo—A Multichannel Digital and Telephone Support Program for People With Chronic Kidney Disease: Feasibility Randomized Controlled Trial
Source: JMIR Form Res. 2023 Nov 23;7:e33147. doi: 10.2196/33147 (PMC10704307; doi:10.2196/33147)
Supplement: Multimedia Appendix 2 [file formative_v7i1e33147_app2.pdf]

WELCOME : SARAH

ckd
CARE.KNOW.DO

LOG OUT | MY PROFILE

Search

HOME | ABOUT CKD | MIND MATTERS | MEDICINE MATTERS | LIFESTYLE MATTERS | SUPPORT MATTERS | MY DATA

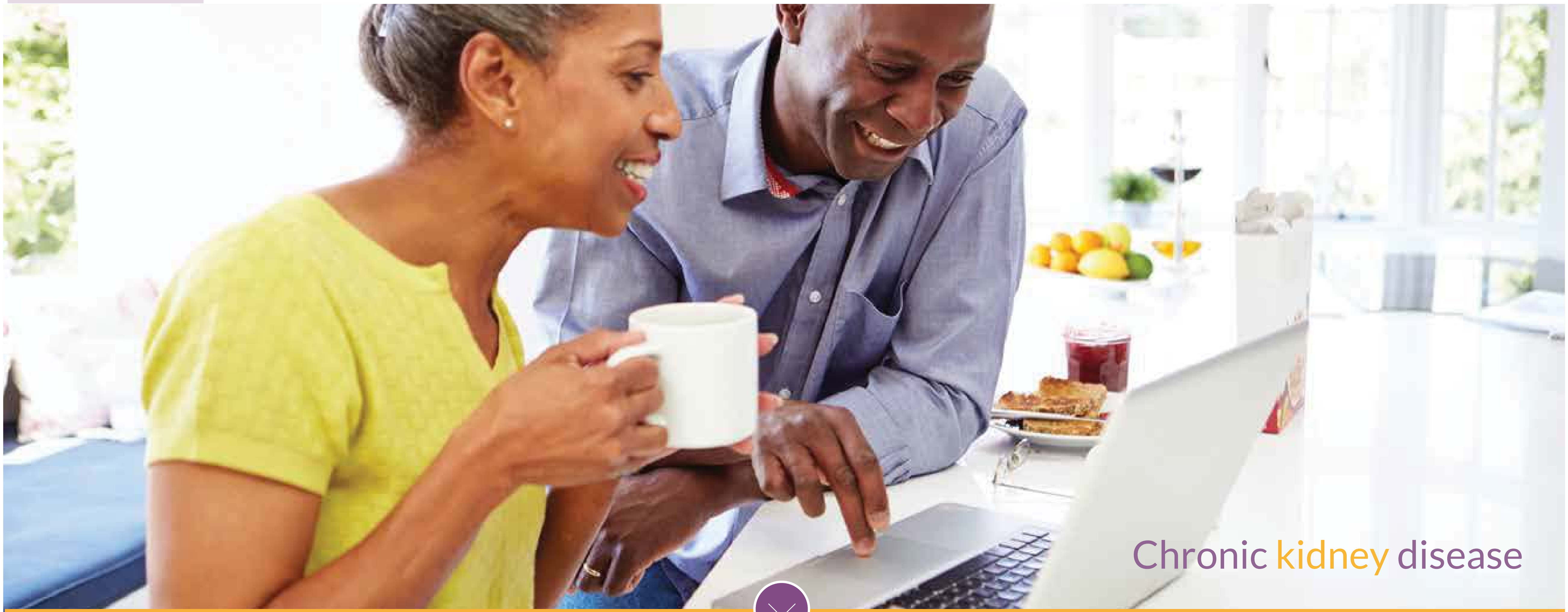

LIVE.LIFE.WELL

ACCESSING SUPPORT HELPED ME BEAT ANXIETY

VIEW AMANDA'S STORY

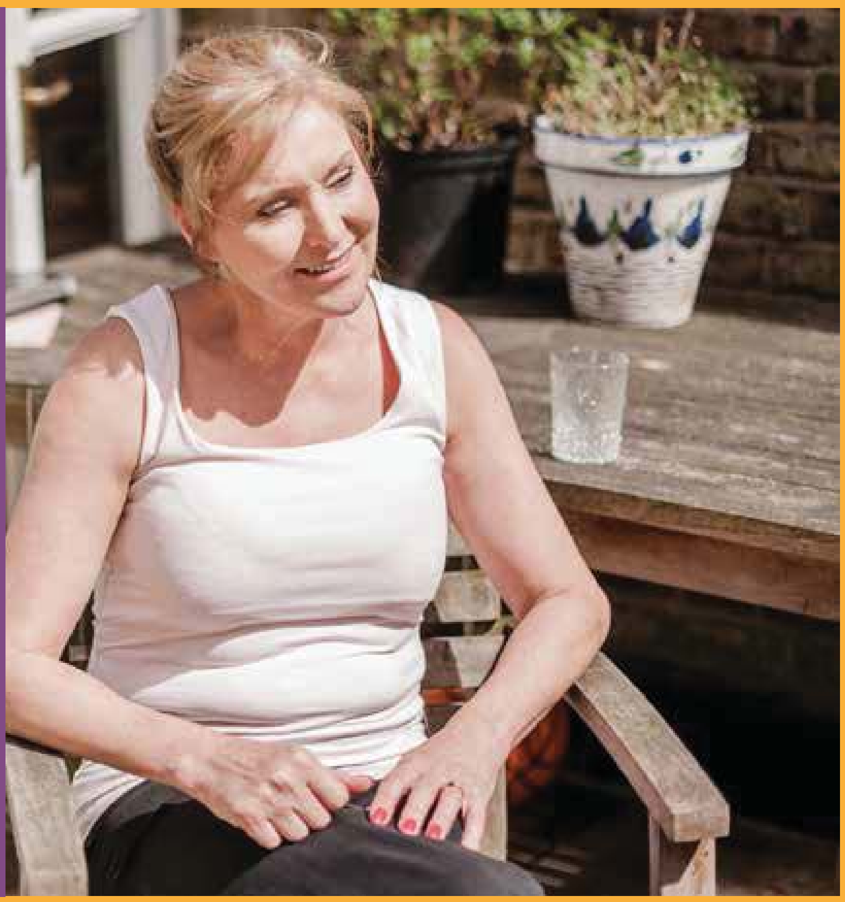

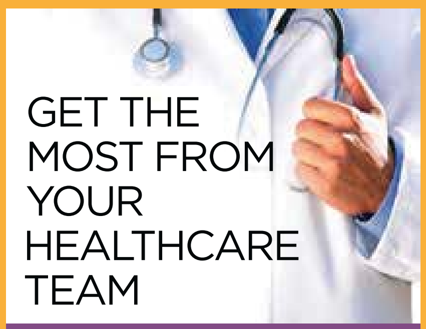

Your must have hints & tips >

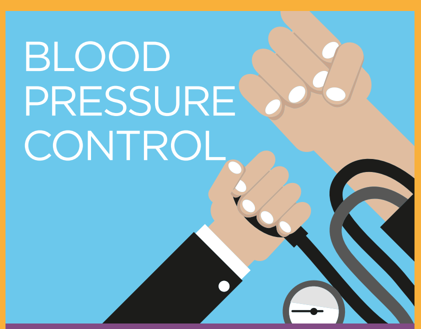

Your need to know guide >

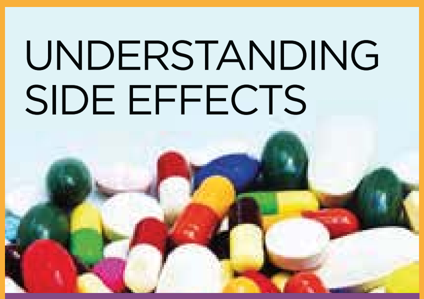

Dr Clarke answers your questions >

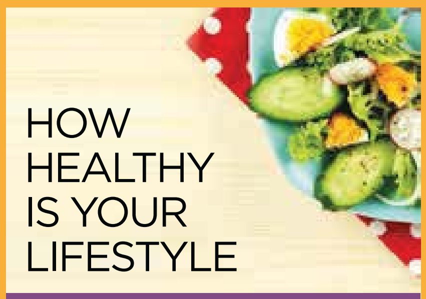

Take the 5-minute test >

BACK.TO.TOP

MIND MATTERS

LEARN MORE >

MEDICINE MATTERS

LEARN MORE >

LIFESTYLE MATTERS

LEARN MORE >

SUPPORT MATTERS

LEARN MORE >

© 2014 Group. All Rights Reserved.

About chronic kidney disease

Chronic kidney disease: some key things you should know

Chronic kidney disease is a long-term condition where the kidneys stop working properly.

- Having kidney disease can mean your kidneys aren't able to do their jobs and waste products build up in your blood, making you feel ill.
- Kidney disease can have different causes but one of the most common is high blood pressure.
- Over time, kidney disease can further raise your risk of experiencing high blood pressure and cardiovascular disease, which includes all diseases that affect the heart and blood vessels, such as heart attacks, angina and strokes. High blood pressure can then cause more damage to the kidneys.
- Managing blood pressure is the best way to stop kidney disease getting worse, no matter what caused it in the first place. You'll be prescribed medicine to help lower your blood pressure, but it's also important to manage it yourself through a healthy diet and lifestyle.

How your kidneys work

Your kidneys, which are about the size of your fist, sit either side of your body, just below your ribcage. Their job is to:

- filter waste products out of your blood, turning them into urine to be taken out of your body
- maintain a healthy blood pressure by constantly adjusting the volume of blood in the body
- keep the right balance of chemicals in your body, helping your heart and muscles to work well
- make the form of vitamin D that keeps your bones healthy
- make a substance that helps to produce red blood cells.

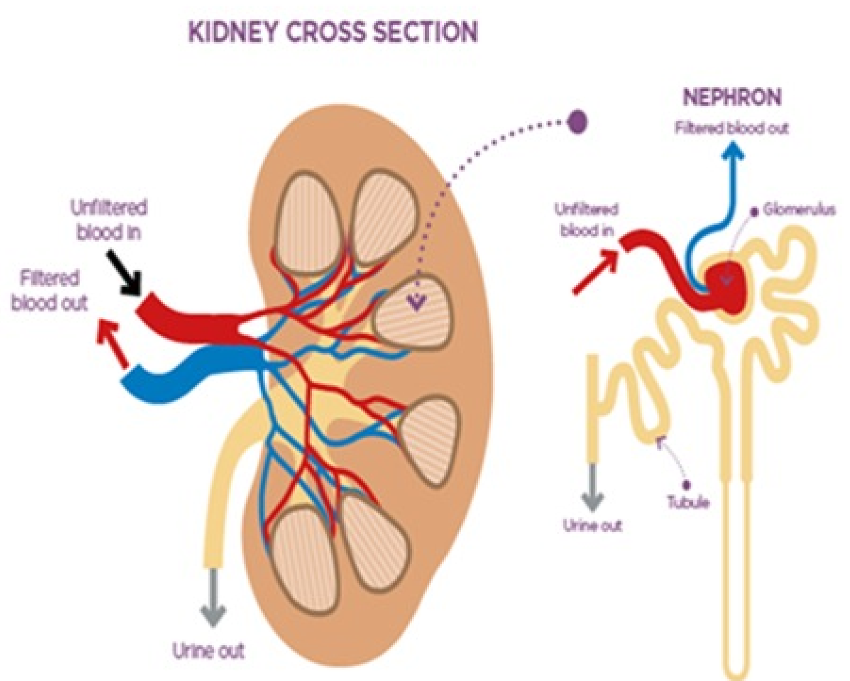

What are the symptoms of kidney disease?

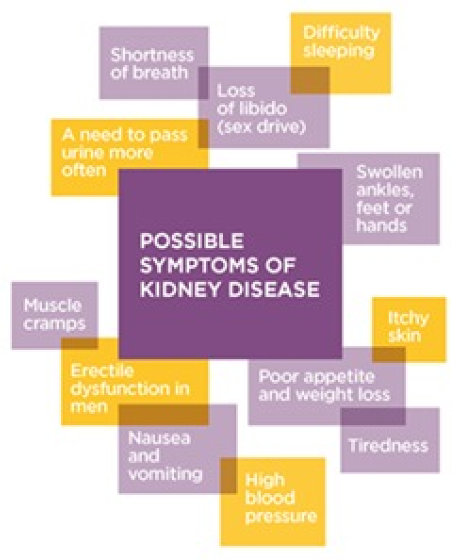

In the early stages of chronic kidney disease, it's unlikely that you'll have any symptoms. Changes in kidney function may only be picked up in blood or urine tests. But if the disease gets worse, waste products build up in your blood, which can make you feel unwell. You may also have symptoms as a result of other problems caused by kidney damage, such as symptoms of anaemia – for example, fatigue - because your kidneys aren't producing enough of the substance that makes red blood cells.

What causes kidney disease?

There are many causes of kidney disease but in the UK the two most common are high blood pressure (also known as hypertension) and diabetes mellitus.

High blood pressure puts strain on the small blood vessels in the kidneys, which stops the kidneys being able to remove waste. Diabetes mellitus can cause high levels of blood sugar. High blood sugar puts strain on the kidneys, making them filter too much blood, and over time, this can damage them.

Damage to your kidneys results in extra fluid in your blood, which pushes your blood pressure even higher. In this way, high blood pressure can cause kidney disease, but kidney disease can also cause high blood pressure. This is why it's vital to manage blood pressure if you have kidney disease. Although there's no cure, keeping your blood pressure at a healthy level is the best way to stop kidney disease getting worse.

You can see the kidney disease-blood pressure cycle in more detail here.

It's also important to note that high blood pressure and high blood sugar as a result of diabetes can both lead to other health problems, such as cardiovascular disease. This includes conditions that affect the heart and blood vessels, such as heart attacks, angina and stroke.

There are some less common causes of chronic kidney disease such as inflammation or infection of the kidneys, blockages to the kidneys caused by other diseases, such as prostate cancer or kidney stones. Talk to your doctor to find out what caused yours.

How common is kidney disease?

One in 10 people in the UK have kidney disease. It's less common in younger people, but in those aged 75 and above, that number rises to one in two.

Will my kidney disease get worse?

There's no cure for kidney disease but sometimes the kidneys will work well for a long time without getting seriously worse. Fewer than one in 10 people will ever get to the point of needing dialysis or a transplant. The outlook often depends on what caused your kidney disease in the first place, so your doctor will be able to talk to you about your individual situation.

But whatever the cause, high blood pressure increases the chance that kidney disease will get worse, and raises your risk of having a heart attack or stroke – so managing blood pressure it is an important way to help slow the progression of kidney disease and prevent other complications. Your doctor will take your blood pressure regularly, or may suggest you get a home blood pressure monitor so you can check it yourself. You can read more about blood pressure readings here.

The stages of kidney disease

There are five stages of kidney disease. To assess the stage your kidney disease is at, your doctor will regularly measure your eGFR, which stands for estimated glomerular filtration rate. This test measures levels of a substance called creatinine, a waste product in your blood that comes from the muscles. It's only removed by your kidneys, so the lower your kidney function, the more creatinine there will be in your blood.

Kidney Function Stages

Stage 1 | Stage 2 | Stage 3A + 3B | Stage 4 | Stage 5

eGFR 90+ ml/min/1.73m2

What this means

Your kidney function is normal, meaning kidneys are still cleaning the blood normally, but tests suggest early signs of kidney disease, for example, showing protein and/or blood is starting to leak through your kidney's filters so it ends up in your urine.

Your treatment

- You'll be monitored regularly
- It's vital to control your blood pressure so it doesn't speed up kidney damage. You might be given medicine to help control it.
- You may be referred to a kidney specialist if your kidney function keeps getting worse.

What you can do

You may be given lifestyle advice to help control your blood pressure, slow the progression of kidney disease and reduce your risk of having a heart attack or stroke. You might be advised to:

- lose weight if you need to
- stop smoking
- reduce salt
- have a healthy diet

Popular Link

How low mood develops
Learn how your thoughts and behaviour can affect the way that you feel.

Manage your medicines
Tips for getting into a routine for your medicine.

Getting the help you need
Tap into support from your family, friends and healthcare team.

Managing your mood
Simple steps that you can take to feel more positive and in control.

Look after yourself
Understanding the link between kidney disease and your lifestyle.

Make a healthy lifestyle work for you
How having goals can help you fit in healthy eating and activity as part of your everyday life.

Positive lifestyle steps you can follow
Having a healthy lifestyle is very important.

Q&A: Your side effects
A kidney nurse answers your questions on blood pressure medicines.

Blood pressure-kidney disease cycle
Find out how the two conditions go hand-in-hand.

© Atlantis Healthcare 2016

Mind matters

This module is designed to help you with any difficult feelings you may have about your condition. You can:

- read first-hand accounts from other people with chronic kidney disease experience
- learn what support is available
- find simple, practical ways to help yourself feel better.

After working through this module, you'll have lots of tools for dealing with the different emotions that you may experience.

How to use this module:

Work through the sections from the beginning, or go directly to the content that you think will be most helpful to you at the moment by clicking on the links below.

In this module:

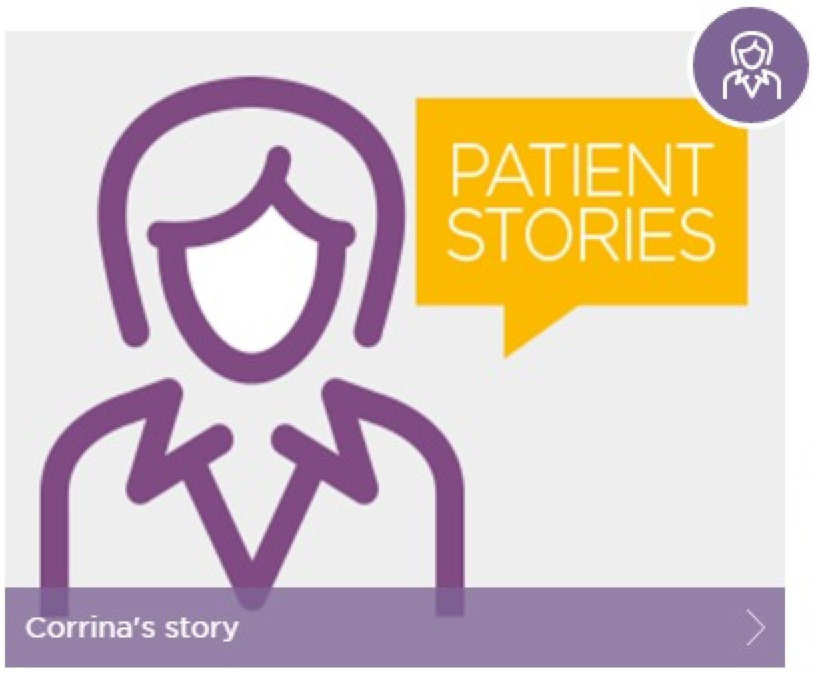

Corrina's story

VIEWED

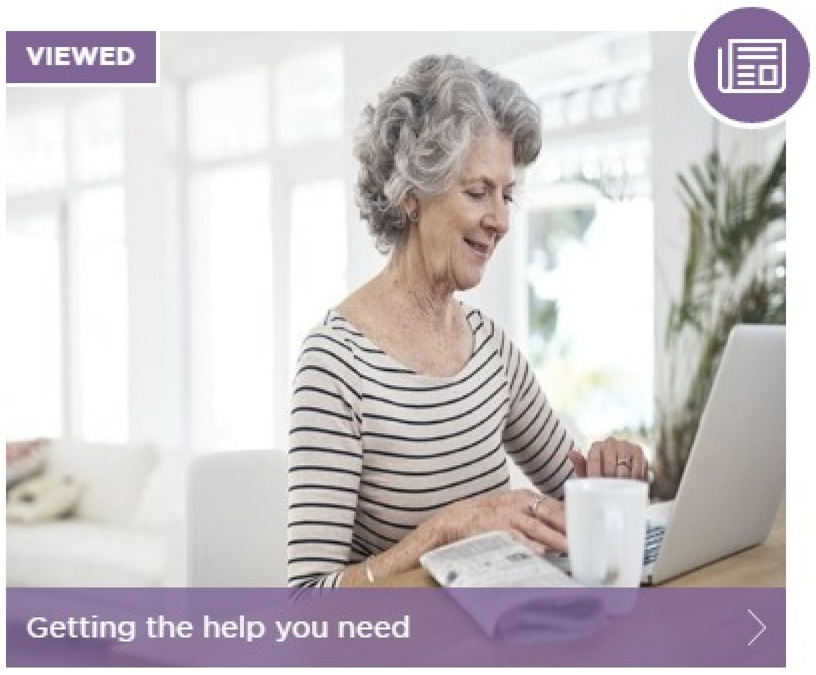

Getting the help you need

VIEWED

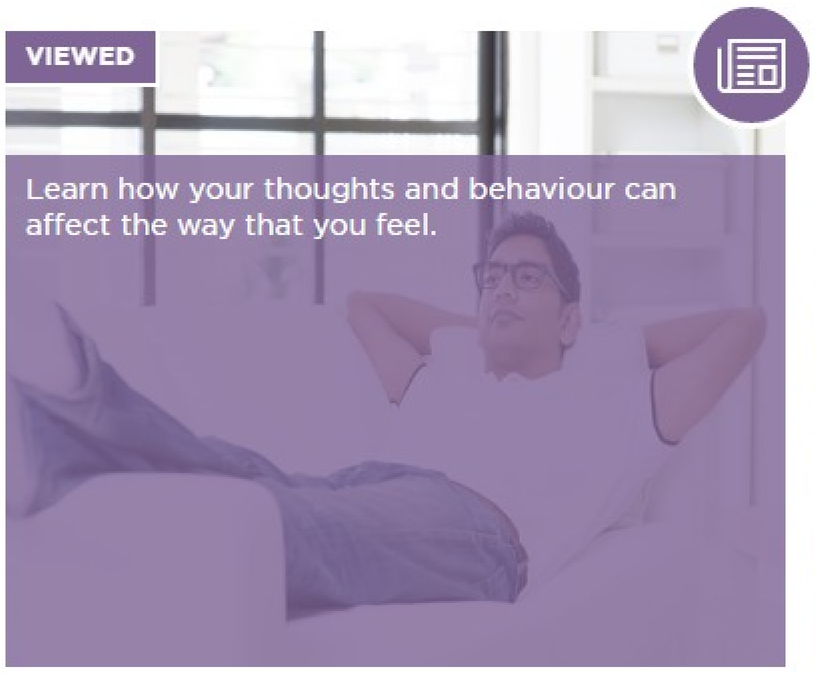

Learn how your thoughts and behaviour can affect the way that you feel.

VIEWED

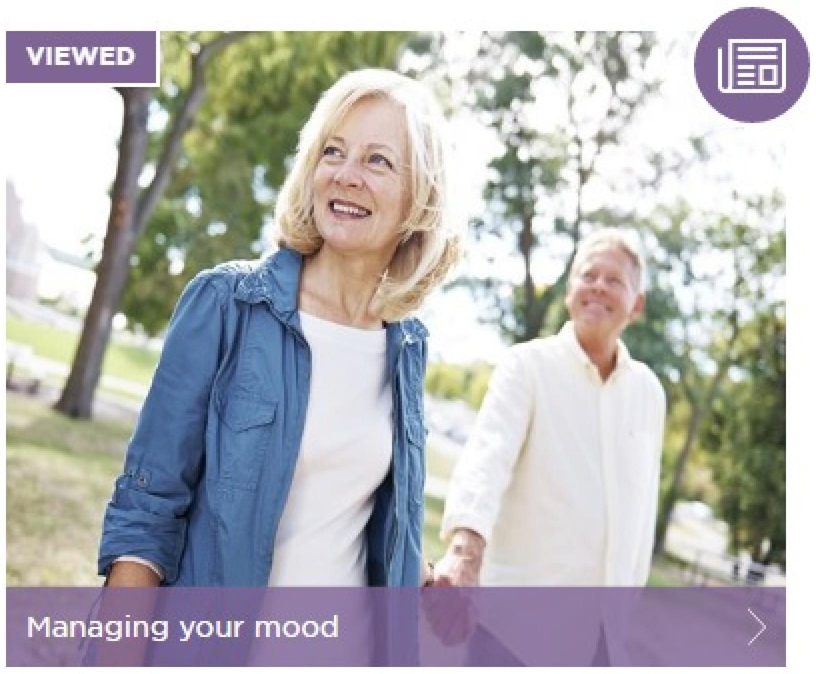

Managing your mood

My Action Plan

View

You

View

Medicine challenges

View

© Atlantis Healthcare 2016

Getting the Help you Need

Low mood can make life feel difficult and joyless. And feeling depressed or anxious can make it harder to look after yourself by taking your medicines as prescribed and following a healthy lifestyle.

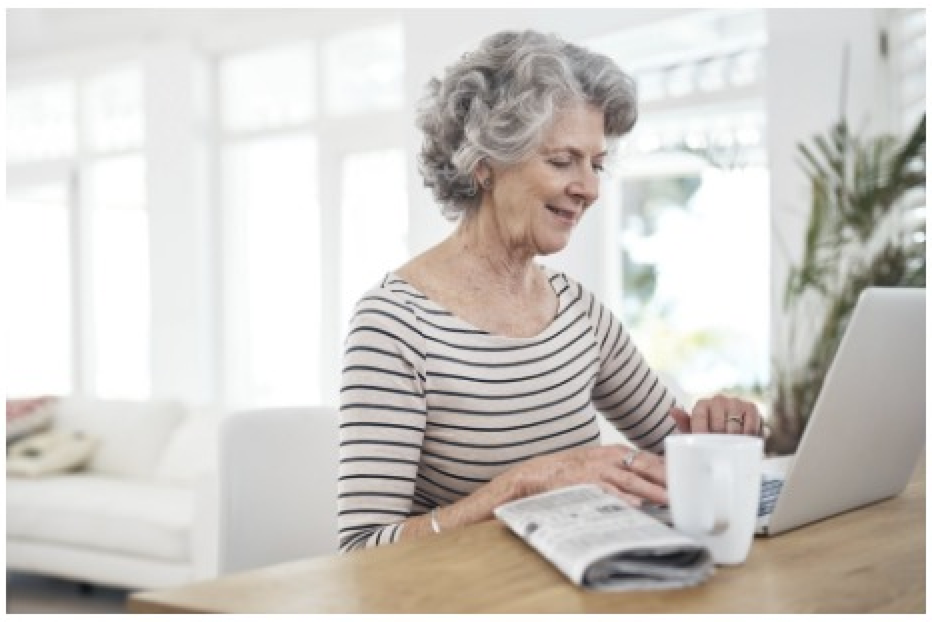

Research shows that people with kidney disease who are also depressed tend to have more complications from their condition and are at higher risk of cardiovascular disease. So it's important to get help if you're affected. The good news is there are different sources of support that can help you to feel better.

Talk to your doctor

If you haven't already spoken to your GP about low mood and anxiety, discuss it with them. They will be able to help you – for example, by referring you for a talking therapy like cognitive behavioural therapy (CBT), which can help to look at negative or unhelpful thoughts and feelings that you may be having. They may also be able to offer practical support to help you manage your kidney disease. For example they might give you guidance on what foods to eat more of, or offer advice on quitting smoking.

You could also talk to your specialist renal team as there may be a clinical psychologist or counsellor within the renal unit you could be referred to.

'It isn't always easy talking about your feelings with your doctor but remember they deal with depression and anxiety all the time. They will want to help you,' says our Health Psychology Specialist, Sumira. 'If you don't feel comfortable talking to your GP, try speaking to another member of your healthcare team, such as your nurse or your consultant, who may be able to write your GP a letter asking them to refer you for therapy.'

Try these conversation starters:

'There's something I would like to speak to you about, but I'm finding it quite difficult.'

'I've been feeling down recently and wondered if we could talk about that.'

Friends and family

'Friends and relatives can provide a listening ear when you're feeling down,' says our Health Psychologist, Sumira. 'And, if depression is making you irritable or quiet, letting them know how you're feeling can help them to be more understanding. They can also help you in practical ways, which may help take the pressure off you. It's natural that you may feel concerned about worrying them, but often friends and family know something's wrong and want to help.'

Try these conversation starters:

'Could we make some time to talk? There's something I'd like to chat to you about.'

'You might have noticed I've been different recently – I think it's because I'm feeling low.'

Charities and support groups

Charities can provide information and tell you how to get help in your area. They can also give you details of support groups, where you can talk to other people who are having similar feelings, whether that's in person or online.

Mind: email info@mind.org.uk or call the helpline on 0300 123 3393
Sane: call the helpline on 0300 304 7000

Popular Links

How low mood develops
Learn how your thoughts and behaviour can affect the way that you feel.

Getting the help you need
Tap into support from your family, friends and healthcare team.

Managing your mood
Simple steps that you can take to feel more positive and in control.

© Atlantis Healthcare 2016

Medication Matters

Are you wondering how your medicine helps your chronic kidney disease? Perhaps you're concerned about side effects or you're finding it hard to take your medicines as prescribed. In this module, you can learn about your medicines and find out how to manage any difficulties you're having. Once you have worked through this module, you will understand how your medicine is helping and you'll feel more confident about taking it.

How to use the module:

Work through the sections from the beginning, or go directly to the content that you think will be most helpful to you at the moment by clicking on the links below.

In this module:

VIEWED

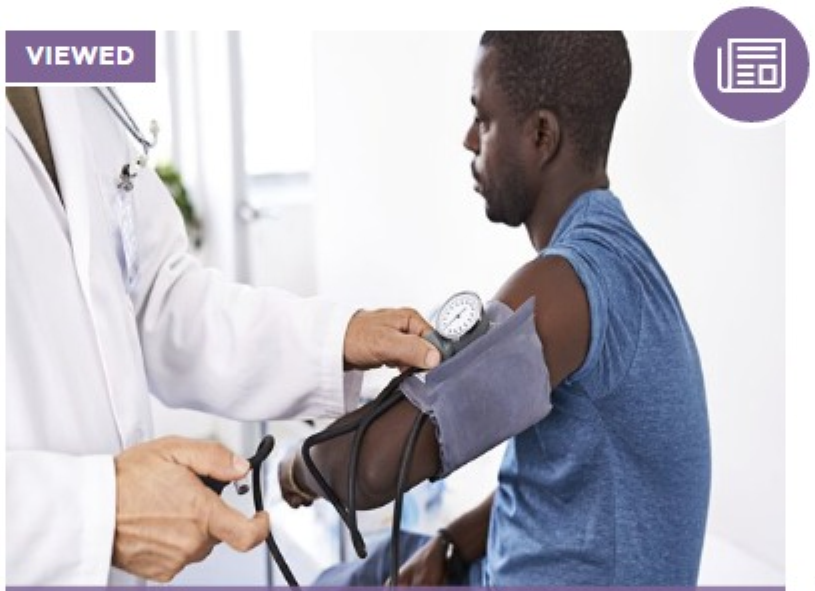

Blood pressure-kidney disease cycle

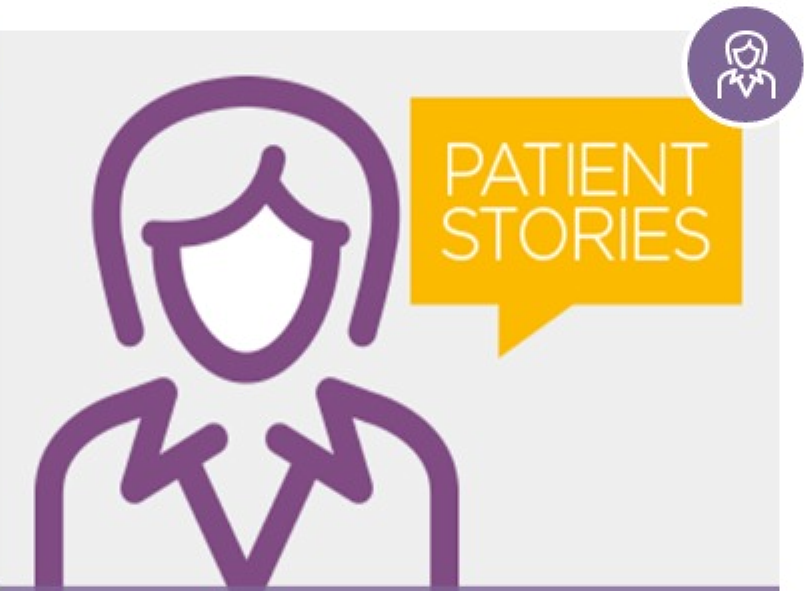

Denny's story

VIEWED

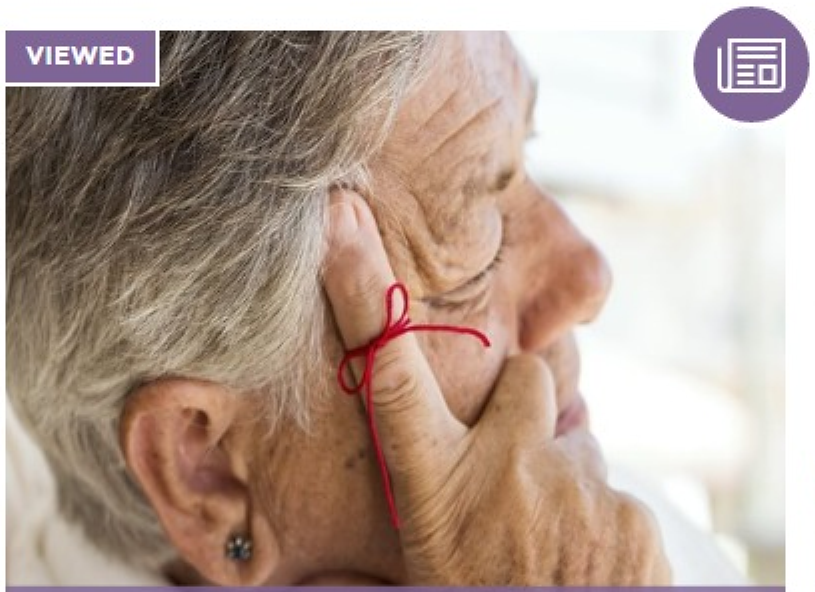

Manage your medicines

VIEWED

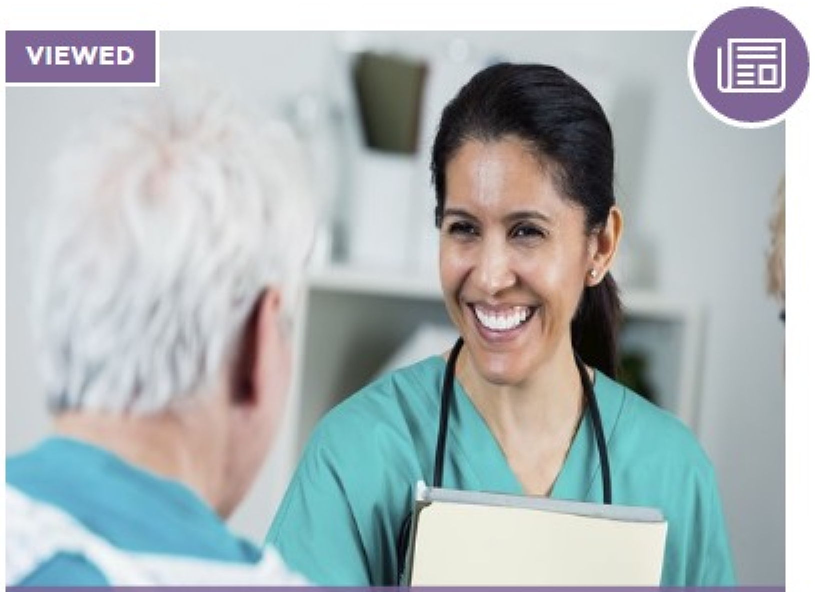

Q&A: Your side effects

Medicine: addressing your concerns

View

Can you change the way that you view your medicine?

View

Medicine challenges

View

© Atlantis Healthcare 2016

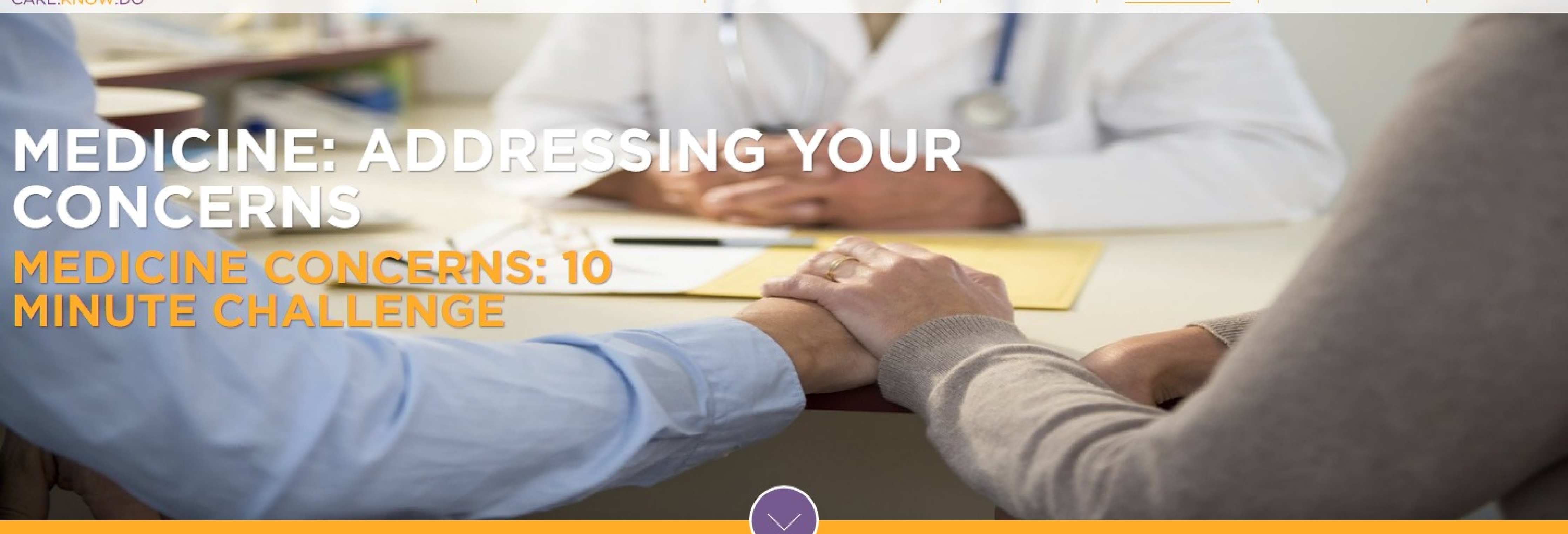

MEDICINE: ADDRESSING YOUR CONCERNS

MEDICINE CONCERNS: 10 MINUTE CHALLENGE

We all have thoughts that are either untrue or unhelpful, because they make us feel anxious and don't solve problems. Living with a long-term condition like chronic kidney disease can be difficult and it's normal to have these unhelpful feelings from time to time. But there are ways to challenge and change these thoughts.

This 10 minute challenge will help you to think about any concerns that you may have about your medicine. This may come from past experiences, things you have heard or perhaps information you have read.

QUESTION 1

When you think 'I am concerned about taking my medicine', how do you feel? (Tick all that apply)

Enter other feelings if needed. Press enter to submit.

- [ ] SAD
- [x] CONFUSED
- [x] ANXIOUS
- [ ] HOPEFUL
- [ ] FEARFUL
- [ ] POSITIVE
- [ ] CONFIDENT
- [ ] CERTAIN
- [ ] RELAXED
- [ ] HAPPY
- [ ] WORRIED
- [ ] ANGRY
- [ ] IRRITATED
- [ ] MOTIVATED
- [ ] DISAPPOINTED
- [ ] FRUSTRATED
- [ ] IN CONTROL

TYPE OTHER FEELING IF NEEDED

Press enter to submit

QUESTION 2

What evidence is there that you should be concerned about taking your medicine?

THINK OF YOUR TOP THREE REASONS

Side effects information in the package leaflet

WRITE YOUR 'EVIDENCE FOR TAKING YOUR MEDICINE' IN HERE

WRITE YOUR 'EVIDENCE FOR TAKING YOUR MEDICINE' IN HERE

FOR EXAMPLE

'I read on the internet that the medicine can cause lots of side effects', or 'I've heard that my medicine is addictive'

QUESTION 3

Now think of reasons you shouldn't be concerned about taking your medicine.

THINK OF YOUR TOP THREE REASONS

My doctor is educated in the best treatments for people with kidney problems

WRITE YOUR 'EVIDENCE AGAINST TAKING YOUR MEDICINE' IN HERE

WRITE YOUR 'EVIDENCE AGAINST TAKING YOUR MEDICINE' IN HERE

FOR EXAMPLE

'I haven't had many side effects so far', or 'Since I started taking my medicine, I'm feeling better.'

QUESTION 4

The next step is to balance the pros and cons, or your 'evidence for' and 'evidence against' the belief that you should be concerned about taking your medicine.

EVIDENCE FOR	HOW MUCH DO YOU BELIEVE THIS?	EVIDENCE AGAINST	HOW MUCH DO YOU BELIEVE THIS?
Side effects information in the package leaflet	Please select a number<br>0 1 2 3 4 5 (4 selected)<br>Not at all … Very strongly	My doctor is educated in the best treatments for people with kidney problems	Please select a number<br>0 1 2 3 4 5 (4 selected)<br>Not at all … Very strongly

QUESTION 5

Did the evidence stack up for taking your medicine? Can you think of another, more balanced way of thinking about it now? Write down this thought in the space below.

Record this thought in the space below.

My doctor should recommend the best option - and all medicines have some risk of side effects.

QUESTION 6

Having weighed up the evidence, how do you feel now? (select all that apply)

Enter other feelings if needed. Press enter to submit.

- [ ] SAD
- [ ] CONFUSED
- [ ] ANXIOUS
- [ ] HOPEFUL
- [ ] FEARFUL
- [ ] POSITIVE
- [ ] CONFIDENT
- [ ] CERTAIN
- [ ] RELAXED
- [ ] HAPPY
- [ ] WORRIED
- [ ] ANGRY
- [ ] IRRITATED
- [ ] MOTIVATED
- [ ] DISAPPOINTED
- [ ] FRUSTRATED
- [ ] IN CONTROL

Calm

Press enter to submit

QUESTION 7

Type your next steps here. If you are still worried about taking your medicine, your next steps might be to make a plan for talking to a doctor about this.

For help with planning easy, realistic and achievable steps, use the Care.Know.Do Action Planner.

I will ask my specialist at the next appointment how much risk this medicine has for someone like me.

Print | Save

© Atlantis Healthcare 2016

Homepage > Medicine Matters > Tools > You and your medicine

This challenge will help you think about how you view your medicine. To do this, we are going to ask you about the pros and cons around whether you need to take your medicine. This might come from past experience, things you've heard or perhaps information you've read.

QUESTION 1

When you think 'I don't need to take my medicine', what feelings do you have? (select all that apply)

Enter other feelings if needed Press enter to submit

SAD ☐	CONFUSED ☐	ANXIOUS ☐	HOPEFUL ☐
FEARFUL ☐	POSITIVE ☐	CONFIDENT ☐	CERTAIN ☐
RELAXED ☐	HAPPY ☐	WORRIED ☐	ANGRY ☐
IRRITATED ☐	MOTIVATED ☐	DISAPPOINTED ☐	FRUSTRATED ☐
IN CONTROL ☐			

TYPE OTHER FEELING IF NEEDED

Press enter to submit

QUESTION 2

What evidence do you have to make you think that you don't need to take your medicine?

THINK OF YOUR TOP THREE REASONS

yes

WRITE YOUR 'EVIDENCE FOR TAKING YOUR MEDICINE' IN HERE

WRITE YOUR 'EVIDENCE FOR TAKING YOUR MEDICINE' IN HERE

FOR EXAMPLE

'My doctor has told me that it will help my condition' or 'The medicine has helped other people with kidney disease.'

QUESTION 3

What evidence do you have that your medicine is helping you at the moment?

THINK OF YOUR TOP THREE REASONS

no

WRITE YOUR 'EVIDENCE AGAINST TAKING YOUR MEDICINE' IN HERE

WRITE YOUR 'EVIDENCE AGAINST TAKING YOUR MEDICINE' IN HERE

FOR EXAMPLE

'I haven't noticed any improvement in my symptoms' or 'I don't think this is the right medicine for me.'

QUESTION 4

The next step is to balance the pros and cons, or your 'evidence for' and 'evidence against' the belief that you need to take your medicine.

EVIDENCE FOR	HOW MUCH DO YOU BELIEVE THIS?	EVIDENCE AGAINST	HOW MUCH DO YOU BELIEVE THIS?
yes	Please select a number<br>0 1 2 3 4 5<br>○ ○ ○ ○ ○ ○<br>Not at all Very strongly	no	Please select a number<br>0 1 2 3 4 5<br>○ ○ ○ ○ ○ ○<br>Not at all Very strongly

QUESTION 5

Was the evidence in favour of taking your medicine? Can you think of an alternative more balanced way of thinking about your medicine?

Record this thought in the space below.

QUESTION 6

Having weighed up the evidence, how do you feel now? (select all that apply)

Enter other feelings if needed Press enter to submit

SAD ☐	CONFUSED ☐	ANXIOUS ☐	HOPEFUL ☐
FEARFUL ☐	POSITIVE ☐	CONFIDENT ☐	CERTAIN ☐
RELAXED ☐	HAPPY ☐	WORRIED ☐	ANGRY ☐
IRRITATED ☐	MOTIVATED ☐	DISAPPOINTED ☐	FRUSTRATED ☐
IN CONTROL ☐			

TYPE OTHER FEELING IF NEEDED

Press enter to submit

QUESTION 7

Are you still worried about taking your medicine? If so, you could plan a visit to your doctor to discuss these concerns. Type your next steps here.

For help with planning easy, realistic and achievable steps, use the Care.Know.Do Action Planner.

Print Save

© Atlantis Healthcare 2016

ckd

CARE.KNOW.DO

MY SETTINGS | LOG OUT

HOME | ABOUT CARE.KNOW.DO | ABOUT KIDNEY DISEASE | MIND MATTERS | MEDICINE MATTERS | LIFESTYLE MATTERS | MY HEALTH

Homepage › **Lifestyle Matters**

Lifestyle Matters

Leading a healthy lifestyle and taking your medication can help stop your chronic kidney disease getting worse – it can also lower your risk of other serious conditions. The Lifestyle Matters module highlights how the way you live can make a difference, while showing you some simple ways to be healthier day to day. When you've worked through it, you'll feel more confident about making healthier lifestyle changes, from improving your diet to fitting in more activity.

How to use this module

Work through the sections from the beginning, or go directly to the content that you think will be most helpful to you at the moment, by clicking on the links below.

In this module:

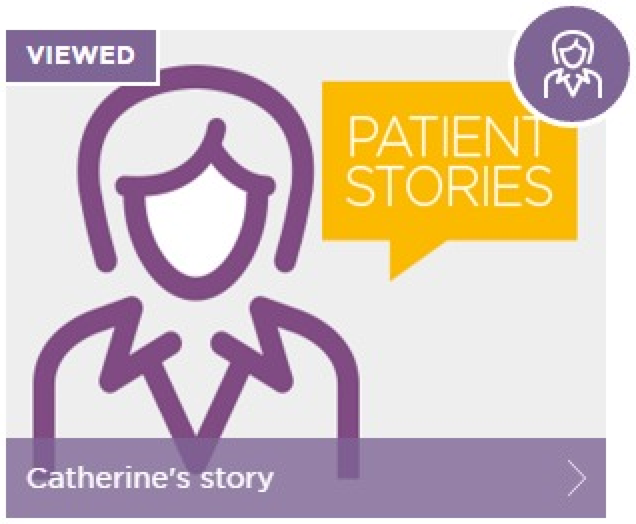

VIEWED

Catherine's story

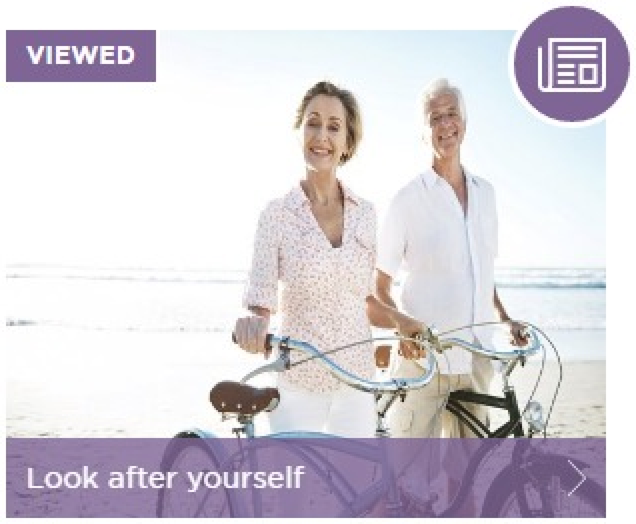

VIEWED

Look after yourself

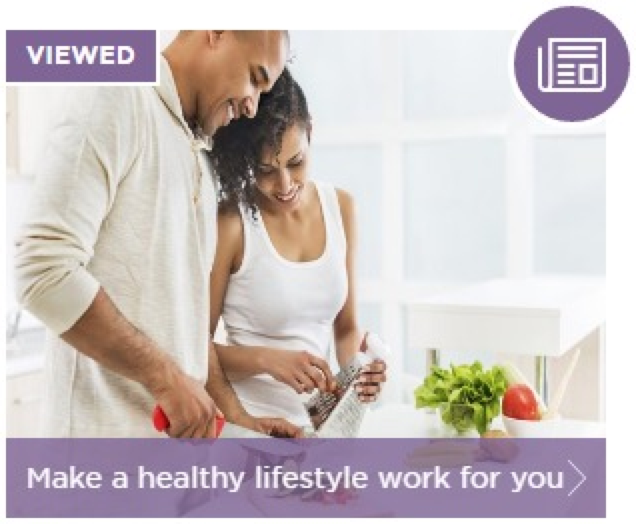

VIEWED

Make a healthy lifestyle work for you

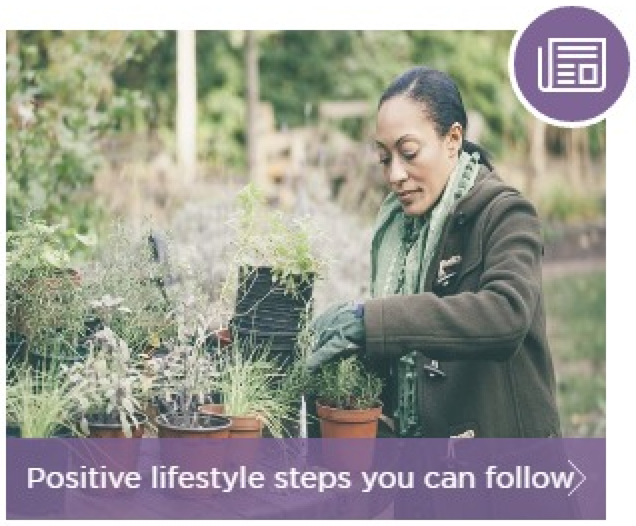

Positive lifestyle steps you can follow

Lifestyle challenges

View

Lifestyle: addressing your concerns

View

Your personal action plan

View

© Atlantis Healthcare 2016
