# Supplementary material for: CareKnowDo—A Multichannel Digital and Telephone Support Program for People With Chronic Kidney Disease: Feasibility Randomized Controlled Trial
Source: JMIR Form Res. 2023 Nov 23;7:e33147. doi: 10.2196/33147 (PMC10704307; doi:10.2196/33147)
Supplement: Multimedia Appendix 3 [file formative_v7i1e33147_app3.pdf]

CONSORT-EHEALTH (V 1.6.1) - Submission/Publication Form

The CONSORT-EHEALTH checklist is intended for authors of randomized trials evaluating web-based and Internet-based applications/interventions, including mobile interventions, electronic games (incl multiplayer games), social media, certain telehealth applications, and other interactive and/or networked electronic applications. Some of the items (e.g. all subitems under item 5 - description of the intervention) may also be applicable for other study designs.

The goal of the CONSORT EHEALTH checklist and guideline is to be
a) a guide for reporting for authors of RCTs,
b) to form a basis for appraisal of an ehealth trial (in terms of validity)

CONSORT-EHEALTH items/subitems are MANDATORY reporting items for studies published in the Journal of Medical Internet Research and other journals / scientific societies endorsing the checklist.

Items numbered 1., 2., 3., 4a., 4b etc are original CONSORT or CONSORT-NPT (non-pharmacologic treatment) items.
Items with Roman numerals (i., ii, iii, iv etc.) are CONSORT-EHEALTH extensions/clarifications.

As the CONSORT-EHEALTH checklist is still considered in a formative stage, we would ask that you also RATE ON A SCALE OF 1-5 how important/useful you feel each item is FOR THE PURPOSE OF THE CHECKLIST and reporting guideline (optional).

Mandatory reporting items are marked with a red *.
In the textboxes, either copy & paste the relevant sections from your manuscript into this form - please include any quotes from your manuscript in QUOTATION MARKS,
or answer directly by providing additional information not in the manuscript, or elaborating on why the item was not relevant for this study.

YOUR ANSWERS WILL BE PUBLISHED AS A SUPPLEMENTARY FILE TO YOUR PUBLICATION IN JMIR AND ARE CONSIDERED PART OF YOUR PUBLICATION (IF ACCEPTED).
Please fill in these questions diligently. Information will not be copyedited, so please use proper spelling and grammar, use correct capitalization, and avoid abbreviations.

DO NOT FORGET TO SAVE AS PDF _AND_ CLICK THE SUBMIT BUTTON SO YOUR ANSWERS ARE IN OUR DATABASE !!!

Citation Suggestion (if you append the pdf as Appendix we suggest to cite this paper in the caption):
Eysenbach G, CONSORT-EHEALTH Group
CONSORT-EHEALTH: Improving and Standardizing Evaluation Reports of Web-based and Mobile Health Interventions
J Med Internet Res 2011;13(4):e126

URL: http://www.jmir.org/2011/4/e126/
doi: 10.2196/jmir.1923
PMID: 22209829

jonathan.reston@atlantishealthcare.com (not shared) Switch account

Draft not saved

* Required

Your name *

First Last

Jonathan Reston

Primary Affiliation (short), City, Country *

University of Toronto, Toronto, Canada

Atlantis Health & King's College London

Your e-mail address *

abc@gmail.com

jonathan.reston@atlantishealth.com

Title of your manuscript *

Provide the (draft) title of your manuscript.

CareKnowDo – A Multichannel Digital Support Program for People With Chronic Kidney Disease: A Feasibility Randomized Controlled Trial

Name of your App/Software/Intervention *

If there is a short and a long/alternate name, write the short name first and add the long name in brackets.

CareKnowDo

Evaluated Version (if any)

e.g. "V1", "Release 2017-03-01", "Version 2.0.27913"

Your answer

Language(s) *

What language is the intervention/app in? If multiple languages are available, separate by comma (e.g. "English, French")

English

URL of your Intervention Website or App

e.g. a direct link to the mobile app on app in appstore (itunes, Google Play), or URL of the website. If the intervention is a DVD or hardware, you can also link to an Amazon page.

https://www.hra.nhs.uk/planning-and-improving-research/application-summaries/research-s

URL of an image/screenshot (optional)

Your answer

Accessibility *

Can an enduser access the intervention presently?

- ○ access is free and open
- ○ access only for special usergroups, not open
- ○ access is open to everyone, but requires payment/subscription/in-app purchases
- ○ app/intervention no longer accessible
- ◉ Other: Website is not currently in use by endusers until further trials

Primary Medical Indication/Disease/Condition *

e.g. "Stress", "Diabetes", or define the target group in brackets after the condition, e.g. "Autism (Parents of children with)", "Alzheimers (Informal Caregivers of)"

Chronic Kidney Disease

Primary Outcomes measured in trial *

comma-separated list of primary outcomes reported in the trial

Feasibility, uptake, randomisation, survey resp[illegible]

Secondary/other outcomes

Are there any other outcomes the intervention is expected to affect?

Blood pressure, beliefs about illness (B-IPQ), beliefs about treatment (BMQ)

Recommended "Dose" *

What do the instructions for users say on how often the app should be used?

- ○ Approximately Daily
- ○ Approximately Weekly
- ○ Approximately Monthly
- ○ Approximately Yearly
- ○ "as needed"
- ◉ Other: As a tailored intervention, users will recieve differing frequencies of su

Approx. Percentage of Users (starters) still using the app as recommended after 3 months *

- ○ unknown / not evaluated
- ○ 0-10%
- ○ 11-20%
- ○ 21-30%
- ◉ 31-40%
- ○ 41-50%
- ○ 51-60%
- ○ 61-70%
- ○ 71%-80%
- ○ 81-90%
- ○ 91-100%
- ○ Other:

Overall, was the app/intervention effective? *

- ( ) yes: all primary outcomes were significantly better in intervention group vs control
- ( ) partly: SOME primary outcomes were significantly better in intervention group vs control
- ( ) no statistically significant difference between control and intervention
- ( ) potentially harmful: control was significantly better than intervention in one or more outcomes
- ( ) inconclusive: more research is needed
- (x) Other: The intervention was found to be feasible, and areas for iteration prior

Article Preparation Status/Stage *

At which stage in your article preparation are you currently (at the time you fill in this form)

- ( ) not submitted yet - in early draft status
- ( ) not submitted yet - in late draft status, just before submission
- ( ) submitted to a journal but not reviewed yet
- (x) submitted to a journal and after receiving initial reviewer comments
- ( ) submitted to a journal and accepted, but not published yet
- ( ) published
- ( ) Other:

Journal *

If you already know where you will submit this paper (or if it is already submitted), please provide the journal name (if it is not JMIR, provide the journal name under "other")

- ○ not submitted yet / unclear where I will submit this
- ○ Journal of Medical Internet Research (JMIR)
- ○ JMIR mHealth and UHealth
- ○ JMIR Serious Games
- ○ JMIR Mental Health
- ○ JMIR Public Health
- ◉ JMIR Formative Research
- ○ Other JMIR sister journal
- ○ Other:

Is this a full powered effectiveness trial or a pilot/feasibility trial? *

- ◉ Pilot/feasibility
- ○ Fully powered

Manuscript tracking number *

If this is a JMIR submission, please provide the manuscript tracking number under "other" (The ms tracking number can be found in the submission acknowledgement email, or when you login as author in JMIR. If the paper is already published in JMIR, then the ms tracking number is the four-digit number at the end of the DOI, to be found at the bottom of each published article in JMIR)

- ○ no ms number (yet) / not (yet) submitted to / published in JMIR
- ◉ Other: JFR ms#33147

TITLE AND ABSTRACT

1a) TITLE: Identification as a randomized trial in the title

1a) Does your paper address CONSORT item 1a? *

I.e does the title contain the phrase "Randomized Controlled Trial"? (if not, explain the reason under "other")

- (x) yes
- ( ) Other:

1a-i) Identify the mode of delivery in the title

Identify the mode of delivery. Preferably use “web-based” and/or “mobile” and/or “electronic game” in the title. Avoid ambiguous terms like “online”, “virtual”, “interactive”. Use “Internet-based” only if Intervention includes non-web-based Internet components (e.g. email), use “computer-based” or “electronic” only if offline products are used. Use “virtual” only in the context of “virtual reality” (3-D worlds). Use “online” only in the context of “online support groups”. Complement or substitute product names with broader terms for the class of products (such as “mobile” or “smart phone” instead of “iphone”), especially if the application runs on different platforms.

	1	2	3	4	5	
subitem not at all important	( )	( )	( )	(x)	( )	essential

Clear selection

Does your paper address subitem 1a-i? *

Copy and paste relevant sections from manuscript title (include quotes in quotation marks "like this" to indicate direct quotes from your manuscript), or elaborate on this item by providing additional information not in the ms, or briefly explain why the item is not applicable/relevant for your study

"Multichannel Digital and Telephone"

1a-ii) Non-web-based components or important co-interventions in title

Mention non-web-based components or important co-interventions in title, if any (e.g., “with telephone support”).

	1	2	3	4	5	
subitem not at all important	○	○	○	●	○	essential

Clear selection

Does your paper address subitem 1a-ii?

Copy and paste relevant sections from manuscript title (include quotes in quotation marks "like this" to indicate direct quotes from your manuscript), or elaborate on this item by providing additional information not in the ms, or briefly explain why the item is not applicable/relevant for your study

Multichannel Digital and Telephone"

1a-iii) Primary condition or target group in the title

Mention primary condition or target group in the title, if any (e.g., “for children with Type I Diabetes”) Example: A Web-based and Mobile Intervention with Telephone Support for Children with Type I Diabetes: Randomized Controlled Trial

	1	2	3	4	5	
subitem not at all important	○	○	○	○	●	essential

Clear selection

Does your paper address subitem 1a-iii? *

Copy and paste relevant sections from manuscript title (include quotes in quotation marks "like this" to indicate direct quotes from your manuscript), or elaborate on this item by providing additional information not in the ms, or briefly explain why the item is not applicable/relevant for your study

"for People With Chronic Kidney Disease"

1b) ABSTRACT: Structured summary of trial design, methods, results, and conclusions

NPT extension: Description of experimental treatment, comparator, care providers, centers, and blinding status.

1b-i) Key features/functionalities/components of the intervention and comparator in the METHODS section of the ABSTRACT

Mention key features/functionalities/components of the intervention and comparator in the abstract. If possible, also mention theories and principles used for designing the site. Keep in mind the needs of systematic reviewers and indexers by including important synonyms. (Note: Only report in the abstract what the main paper is reporting. If this information is missing from the main body of text, consider adding it)

	1	2	3	4	5	
subitem not at all important	○	○	○	◉	○	essential

Clear selection

Does your paper address subitem 1b-i? *

Copy and paste relevant sections from the manuscript abstract (include quotes in quotation marks "like this" to indicate direct quotes from your manuscript), or elaborate on this item by providing additional information not in the ms, or briefly explain why the item is not applicable/relevant for your study

Objectives:
The aim of CareKnowDo was to assess the feasibility of rolling out a digital self-management support and adherence program integrated with a patient-facing electronic health record, Patient View (PV).

Methods:
A two arm, parallel, individual-level pragmatic feasibility pilot randomized controlled trial (RCT), running in two National Health Service (NHS) sites in the UK. Sixty-one patients with CKD were randomized 1:1 into two groups and provided with either a new tailored, digital and telephone support program (CareKnowDo, n = 31) integrated with PV, or standard care (PV alone, n = 30).

1b-ii) Level of human involvement in the METHODS section of the ABSTRACT

Clarify the level of human involvement in the abstract, e.g., use phrases like “fully automated” vs. “therapist/nurse/care provider/physician-assisted” (mention number and expertise of providers involved, if any). (Note: Only report in the abstract what the main paper is reporting. If this information is missing from the main body of text, consider adding it)

	1	2	3	4	5	
subitem not at all important	○	○	○	●	○	essential

Clear selection

Does your paper address subitem 1b-ii?

Copy and paste relevant sections from the manuscript abstract (include quotes in quotation marks "like this" to indicate direct quotes from your manuscript), or elaborate on this item by providing additional information not in the ms, or briefly explain why the item is not applicable/relevant for your study

"Sixty-one patients with CKD were randomized 1:1 into two groups and provided with either a new tailored, digital and telephone support program"

1b-iii) Open vs. closed, web-based (self-assessment) vs. face-to-face assessments in the METHODS section of the ABSTRACT

Mention how participants were recruited (online vs. offline), e.g., from an open access website or from a clinic or a closed online user group (closed usergroup trial), and clarify if this was a purely web-based trial, or there were face-to-face components (as part of the intervention or for assessment). Clearly say if outcomes were self-assessed through questionnaires (as common in web-based trials). Note: In traditional offline trials, an open trial (open-label trial) is a type of clinical trial in which both the researchers and participants know which treatment is being administered. To avoid confusion, use “blinded” or “unblinded” to indicated the level of blinding instead of “open”, as “open” in web-based trials usually refers to “open access” (i.e. participants can self-enrol). (Note: Only report in the abstract what the main paper is reporting. If this information is missing from the main body of text, consider adding it)

	1	2	3	4	5	
subitem not at all important	○	○	○	◉	○	essential

Clear selection

Does your paper address subitem 1b-iii?

Copy and paste relevant sections from the manuscript abstract (include quotes in quotation marks "like this" to indicate direct quotes from your manuscript), or elaborate on this item by providing additional information not in the ms, or briefly explain why the item is not applicable/relevant for your study

Results:
"Out of 1,392 patients screened in local kidney clinics, 269 met the basic inclusion criteria, the first eligible 61 of whom were recruited to participate in the study. Twenty-three patients (37.7%) completed the final 6-month follow-up web-based survey."

1b-iv) RESULTS section in abstract must contain use data

Report number of participants enrolled/assessed in each group, the use/uptake of the intervention (e.g., attrition/adherence metrics, use over time, number of logins etc.), in addition to primary/secondary outcomes. (Note: Only report in the abstract what the main paper is reporting. If this information is missing from the main body of text, consider adding it)

	1	2	3	4	5	
subitem not at all important	○	○	○	○	◉	essential

Clear selection

Does your paper address subitem 1b-iv?

Copy and paste relevant sections from the manuscript abstract (include quotes in quotation marks "like this" to indicate direct quotes from your manuscript), or elaborate on this item by providing additional information not in the ms, or briefly explain why the item is not applicable/relevant for your study

"Results:

Out of 1,392 patients screened in local kidney clinics, 269 met the basic inclusion criteria, the first eligible 61 of whom were recruited to participate in the study. Twenty-three patients (37.7%) completed the final 6-month follow-up web-based survey. Reasons for attrition are explored. Higher belief in the ability of treatment to control CKD was associated with lower blood pressure at baseline ($r = .52$, $P = 0.005$), and higher perceived understanding of CKD at baseline was associated with lower blood pressure at follow-up ($r = 0.66$, $P < .001$). Beliefs about medicines at baseline were associated with blood pressure at baseline, but not at follow-up. This was true for both concerns about medicines ($r = .58$, $P = .001$) and perceived necessity of medicines ($r = .42$, $P = .03$)."

1b-v) CONCLUSIONS/DISCUSSION in abstract for negative trials

Conclusions/Discussions in abstract for negative trials: Discuss the primary outcome - if the trial is negative (primary outcome not changed), and the intervention was not used, discuss whether negative results are attributable to lack of uptake and discuss reasons. (Note: Only report in the abstract what the main paper is reporting. If this information is missing from the main body of text, consider adding it)

	1	2	3	4	5	
subitem not at all important	○	○	○	◉	○	essential

Clear selection

Does your paper address subitem 1b-v?

Copy and paste relevant sections from the manuscript abstract (include quotes in quotation marks "like this" to indicate direct quotes from your manuscript), or elaborate on this item by providing additional information not in the ms, or briefly explain why the item is not applicable/relevant for your study

"Conclusions:

A digital support program to enhance support for patients with CKD was piloted in two NHS sites, and found to be feasible and acceptable. However, to maximize the effectiveness of the intervention (and of future trials), consideration should be given to the target audience most likely to benefit, as well as how to help them access the program as quickly and easily as possible."

INTRODUCTION

2a) In INTRODUCTION: Scientific background and explanation of rationale

2a-i) Problem and the type of system/solution

Describe the problem and the type of system/solution that is object of the study: intended as stand-alone intervention vs. incorporated in broader health care program? Intended for a particular patient population? Goals of the intervention, e.g., being more cost-effective to other interventions, replace or complement other solutions? (Note: Details about the intervention are provided in “Methods” under 5)

	1	2	3	4	5	
subitem not at all important	○	○	○	○	◉	essential

Clear selection

Does your paper address subitem 2a-i? *

Copy and paste relevant sections from the manuscript (include quotes in quotation marks "like this" to indicate direct quotes from your manuscript), or elaborate on this item by providing additional information not in the ms, or briefly explain why the item is not applicable/relevant for your study

"For example, across common chronic conditions globally, it has been estimated that between 4 and 31% patients never fill their first prescription, and only 50 to 70% of people take their medications regularly (at least 80% of the time)[13]. This appears to be particularly true of asymptomatic conditions, where the perceived need for treatment is low[14]. For example, in hypertension, only 25-64% of people were estimated to be adherent to their prescribed statin treatment[13]."

"CareKnowDo is a multichannel support programme, developed in collaboration with the Renal Association, that aims to address gap, by providing people living with CKD with tailored web, SMS, email, and nurse phone support based around these types of self-management behaviour. It also features integration with PV, so that results can be viewed directly on the CKD site.

As CareKnowDo (with PV integration) was a novel intervention, the core aim of the study was to establish the feasibility of rolling this out in NHS kidney units, establish uptake, and examine facilitators and barriers to inform future intervention and trial design[28]. Trial design and reporting were guided by the Pilot and Feasibility extension of the 2010 CONSORT guidelines[29]."

2a-ii) Scientific background, rationale: What is known about the (type of) system

Scientific background, rationale: What is known about the (type of) system that is the object of the study (be sure to discuss the use of similar systems for other conditions/diagnoses, if appropiate), motivation for the study, i.e. what are the reasons for and what is the context for this specific study, from which stakeholder viewpoint is the study performed, potential impact of findings [2]. Briefly justify the choice of the comparator.

	1	2	3	4	5	
subitem not at all important	○	○	○	◉	○	essential

Clear selection

Does your paper address subitem 2a-ii? *

Copy and paste relevant sections from the manuscript (include quotes in quotation marks "like this" to indicate direct quotes from your manuscript), or elaborate on this item by providing additional information not in the ms, or briefly explain why the item is not applicable/relevant for your study

"PV allows people being treated for kidney disease by the NHS to view the results of their clinical tests, such as blood and urine tests, as well as recent letters from their nephrologist. Part of the rationale for this is supporting people living with CKD to make decisions, and to take actions, that will reduce the likelihood of progression to ESKD. However, PV provides little information about what to do with this information, or how translate it into behaviour change, such as reducing salt intake, adhering to medicines, or increasing physical activity."

2b) In INTRODUCTION: Specific objectives or hypotheses

Does your paper address CONSORT subitem 2b? *

Copy and paste relevant sections from the manuscript (include quotes in quotation marks "like this" to indicate direct quotes from your manuscript), or elaborate on this item by providing additional information not in the ms, or briefly explain why the item is not applicable/relevant for your study

As CareKnowDo (with PV integration) was a novel intervention, the core aim of the study was to establish the feasibility of rolling this out in NHS kidney units, establish uptake, and examine facilitators and barriers to inform future intervention and trial design[28]. Trial design and reporting were guided by the Pilot and Feasibility extension of the 2010 CONSORT guidelines[29].

METHODS

3a) Description of trial design (such as parallel, factorial) including allocation ratio

Does your paper address CONSORT subitem 3a? *

Copy and paste relevant sections from the manuscript (include quotes in quotation marks "like this" to indicate direct quotes from your manuscript), or elaborate on this item by providing additional information not in the ms, or briefly explain why the item is not applicable/relevant for your study

"Randomisation

Participants were randomized 1:1 to intervention or control. A randomization list was generated in R (v3.3.1),[31] using the package randomizeR (v1.4),[32] which was locked and digitally signed prior to study commencement. A separate randomization list was produced for each of the two study sites. Block randomization approach was used, with random sized blocks of 2-4. After patients had consented to participate in the study, the RN sent the patient ID to the study coordinator, where patients were sequentially allocated against this list."

3b) Important changes to methods after trial commencement (such as eligibility criteria), with reasons

Does your paper address CONSORT subitem 3b? *

Copy and paste relevant sections from the manuscript (include quotes in quotation marks "like this" to indicate direct quotes from your manuscript), or elaborate on this item by providing additional information not in the ms, or briefly explain why the item is not applicable/relevant for your study

"Initially, participants in each arm were to be stratified into 'prevalent' and 'incident' patients, with a recruitment target of 30 for each group, (to capture potential differences between these groups. It was purported that the intervention would be particularly valuable to newer patients. Patients were considered 'prevalent; if they:

- Had been invited to attend three nephology outpatient clinics in the last 12 months
- Attended nephrology outpatient clinics at least twice previously

However, due to a lack of patients being recruited in the incident stratum, this stratification requirement was dropped, and the final sample consisted predominantly of prevalent patients (n = 54 out of 61). "

3b-i) Bug fixes, Downtimes, Content Changes

Bug fixes, Downtimes, Content Changes: ehealth systems are often dynamic systems. A description of changes to methods therefore also includes important changes made on the intervention or comparator during the trial (e.g., major bug fixes or changes in the functionality or content) (5-iii) and other "unexpected events" that may have influenced study design such as staff changes, system failures/downtimes, etc. [2].

	1	2	3	4	5	
subitem not at all important	○	○	○	◉	○	essential

Clear selection

Does your paper address subitem 3b-i?

Copy and paste relevant sections from the manuscript (include quotes in quotation marks "like this" to indicate direct quotes from your manuscript), or elaborate on this item by providing additional information not in the ms, or briefly explain why the item is not applicable/relevant for your study

"Figure 2 shows patient flow after randomization. In each arm, roughly half of participants did not complete enrollment onto the intervention after being randomized. Follow-up RN calls revealed that the most common reasons for this were:

- Not receiving a PV login before attempting to enroll
- Losing invitation emails in spam folders
- Not checking emails
- Technical difficulties logging in"

4a) Eligibility criteria for participants

Does your paper address CONSORT subitem 4a? *

Copy and paste relevant sections from the manuscript (include quotes in quotation marks "like this" to indicate direct quotes from your manuscript), or elaborate on this item by providing additional information not in the ms, or briefly explain why the item is not applicable/relevant for your study

"Participants
Inclusion criteria for the study were that all patients should:
• Have a diagnosis of CKD
o Estimated glomerular filtration rate (eGFR) 15–59 on last measurement and/or latest urine albumin creatinine ration (uACR) /urine protein creatinine ratio (uPCR) >29mg/mmol
• Be at least 18 years old
• Be able to read and speak English (as the pilot intervention was in English only)
• Be computer literate, e.g. have their own email address that they use themselves
• Have access to the internet and mobile phone
• Currently being treated with antihypertensive medication
Exclusion criteria were:
• Patients deemed by their clinician to be likely to need kidney replacement therapy (such as dialysis) within the next 6 months
• Patients with severe or profound intellectual impairments and learning difficulties
Initially, participants in each arm were to be stratified into 'prevalent' and 'incident' patients, with a recruitment target of 30 for each group, (to capture potential differences between these groups. It was purported that the intervention would be particularly valuable to newer patients. Patients were considered 'prevalent; if they:
• Had been invited to attend three nephology outpatient clinics in the last 12 months
• Attended nephrology outpatient clinics at least twice previously
However, due to a lack of patients being recruited in the incident stratum, this stratification requirement was dropped, and the final sample consisted predominantly of prevalent patients (n = 54 out of 61).

4a-i) Computer / Internet literacy

Computer / Internet literacy is often an implicit "de facto" eligibility criterion - this should be explicitly clarified.

	1	2	3	4	5	
subitem not at all important	○	○	○	◉	○	essential

Clear selection

Does your paper address subitem 4a-i?

Copy and paste relevant sections from the manuscript (include quotes in quotation marks "like this" to indicate direct quotes from your manuscript), or elaborate on this item by providing additional information not in the ms, or briefly explain why the item is not applicable/relevant for your study

- "Be computer literate, e.g. have their own email address that they use themselves
- Have access to the internet and mobile phone"

4a-ii) Open vs. closed, web-based vs. face-to-face assessments:

Open vs. closed, web-based vs. face-to-face assessments: Mention how participants were recruited (online vs. offline), e.g., from an open access website or from a clinic, and clarify if this was a purely web-based trial, or there were face-to-face components (as part of the intervention or for assessment), i.e., to what degree got the study team to know the participant. In online-only trials, clarify if participants were quasi-anonymous and whether having multiple identities was possible or whether technical or logistical measures (e.g., cookies, email confirmation, phone calls) were used to detect/prevent these.

	1	2	3	4	5	
subitem not at all important	○	○	○	◉	○	essential

Clear selection

Does your paper address subitem 4a-ii? *

Copy and paste relevant sections from the manuscript (include quotes in quotation marks "like this" to indicate direct quotes from your manuscript), or elaborate on this item by providing additional information not in the ms, or briefly explain why the item is not applicable/relevant for your study

"Patients received an invitation to the study two-weeks prior to their next scheduled clinic appointment. The research nurse (RN) followed this up with a phone call one week prior to their clinic appointment to check whether they were interested and to answer any questions. Recruitment took place face-to-face at the clinic during patient’s scheduled appointment, where baseline information was collected. The patient information sheet included information on the process of randomization into one of the two groups, and a broad idea of what the supportive intervention and control group would entail. Neither group were given in-depth details about the contents of CareKnowDo until after randomisation."

4a-iii) Information giving during recruitment

Information given during recruitment. Specify how participants were briefed for recruitment and in the informed consent procedures (e.g., publish the informed consent documentation as appendix, see also item X26), as this information may have an effect on user self-selection, user expectation and may also bias results.

	1	2	3	4	5	
subitem not at all important	○	○	○	◉	○	essential

Clear selection

Does your paper address subitem 4a-iii?

Copy and paste relevant sections from the manuscript (include quotes in quotation marks "like this" to indicate direct quotes from your manuscript), or elaborate on this item by providing additional information not in the ms, or briefly explain why the item is not applicable/relevant for your study

"The patient information sheet included information on the process of randomization into one of the two groups, and a broad idea of what the supportive intervention and control group would entail. Neither group were given in-depth details about the contents of CareKnowDo until after randomisation."

4b) Settings and locations where the data were collected

Does your paper address CONSORT subitem 4b? *

Copy and paste relevant sections from the manuscript (include quotes in quotation marks "like this" to indicate direct quotes from your manuscript), or elaborate on this item by providing additional information not in the ms, or briefly explain why the item is not applicable/relevant for your study

"Data collection and outcomes

Demographic details including age, gender, ethnicity, age of leaving full time education were collected via the CareKnowDo website. A wide range of clinical details collected included CKD stage, number of prescribed medications, comorbidities, eGFR (a measure of kidney function), and HbA1c. Additionally, the following psychological measures were captured through online self-assesmenent:
Beliefs about Medicines Questionnaire (BMQ):34 Based on the Necessity/Concerns Framework of treatment beliefs. This gives an indication of how necessary a person thinks their medicine is (5 items) and how concerned they are about it (5 items).
Brief Illness Perceptions Questionnaire (B-IPQ):35 Based on the Common Sense Model of Illness Representations, this measures patient beliefs about specific aspects of their illness (eg, identity; personal control; treatment control; consequences; emotional impact; timeline; coherence), using single item scales (score range 1-10)
PHQ-9:38 A screening measure of depressive symptoms used in both research, and clinical practice in the UK."

4b-i) Report if outcomes were (self-)assessed through online questionnaires

Clearly report if outcomes were (self-)assessed through online questionnaires (as common in web-based trials) or otherwise.

	1	2	3	4	5	
subitem not at all important	○	○	○	◉	○	essential

Clear selection

Does your paper address subitem 4b-i? *

Copy and paste relevant sections from the manuscript (include quotes in quotation marks "like this" to indicate direct quotes from your manuscript), or elaborate on this item by providing additional information not in the ms, or briefly explain why the item is not applicable/relevant for your study

"Additionally, the following psychological measures were captured through online self-assessment:
Beliefs about Medicines Questionnaire (BMQ):34 Based on the Necessity/Concerns Framework of treatment beliefs. This gives an indication of how necessary a person thinks their medicine is (5 items) and how concerned they are about it (5 items).
Brief Illness Perceptions Questionnaire (B-IPQ):35 Based on the Common Sense Model of Illness Representations, this measures patient beliefs about specific aspects of their illness (eg, identity; personal control; treatment control; consequences; emotional impact; timeline; coherence), using single item scales (score range 1-10)
PHQ-9:38 A screening measure of depressive symptoms used in both research, and clinical practice in the UK."

4b-ii) Report how institutional affiliations are displayed

Report how institutional affiliations are displayed to potential participants [on ehealth media], as affiliations with prestigious hospitals or universities may affect volunteer rates, use, and reactions with regards to an intervention.(Not a required item – describe only if this may bias results)

	1	2	3	4	5	
subitem not at all important	○	○	○	○	○	essential

Does your paper address subitem 4b-ii?

Copy and paste relevant sections from the manuscript (include quotes in quotation marks "like this" to indicate direct quotes from your manuscript), or elaborate on this item by providing additional information not in the ms, or briefly explain why the item is not applicable/relevant for your study

Your answer

5) The interventions for each group with sufficient details to allow replication, including how and when they were actually administered

5-i) Mention names, credential, affiliations of the developers, sponsors, and owners

Mention names, credential, affiliations of the developers, sponsors, and owners [6] (if authors/evaluators are owners or developer of the software, this needs to be declared in a "Conflict of interest" section or mentioned elsewhere in the manuscript).

	1	2	3	4	5	
subitem not at all important	○	○	○	○	○	essential

Does your paper address subitem 5-i?

Copy and paste relevant sections from the manuscript (include quotes in quotation marks "like this" to indicate direct quotes from your manuscript), or elaborate on this item by providing additional information not in the ms, or briefly explain why the item is not applicable/relevant for your study

"PV allows people being treated for kidney disease by the NHS to view the results of their clinical tests, such as blood and urine tests, as well as recent letters from their nephrologist. Part of the rationale for this is supporting people living with CKD to make decisions, and to take actions, that will reduce the likelihood of progression to ESKD. However, PV provides little information about what to do with this information, or how translate it into behaviour change, such as reducing salt intake, adhering to medicines, or increasing physical activity. CareKnowDo is a multichannel support programme, developed by a team of doctoral level psychologists at Atlantis Health, in collaboration with the Renal Association, that aims to address this gap. It does so by providing people living with CKD with tailored web, SMS, email, and nurse phone support based around these types of self-management behaviour. It also features integration with PV, so that results can be viewed directly on the CKD site. "

5-ii) Describe the history/development process

Describe the history/development process of the application and previous formative evaluations (e.g., focus groups, usability testing), as these will have an impact on adoption/use rates and help with interpreting results.

	1	2	3	4	5	
subitem not at all important	○	○	●	○	○	essential

Clear selection

Does your paper address subitem 5-ii?

Copy and paste relevant sections from the manuscript (include quotes in quotation marks "like this" to indicate direct quotes from your manuscript), or elaborate on this item by providing additional information not in the ms, or briefly explain why the item is not applicable/relevant for your study

"Helping patients to be more involved in their own care (self-management) is publicly claimed to be a core principle of the UK's National Health Service (NHS), although widespread implementation of these ideals has been patchy[26]. One service helping to involve patients in the UK is a service called 'Patient View' (PV), which has been in operation since 2004[27]. PV is run by the Renal Association.
PV allows people being treated for kidney disease by the NHS to view the results of their clinical tests, such as blood and urine tests, as well as recent letters from their nephrologist. Part of the rationale for this is supporting people living with CKD to make decisions, and to take actions, that will reduce the likelihood of progression to ESKD. However, PV provides little information about what to do with this information, or how translate it into behaviour change, such as reducing salt intake, adhering to medicines, or increasing physical activity. CareKnowDo is a multichannel support programme, developed by a team of doctoral level psychologists at Atlantis Health, in collaboration with the Renal Association, that aims to address this gap. It does so by providing people living with CKD with tailored web, SMS, email, and nurse phone support based around these types of self-management behaviour. It also features integration with PV, so that results can be viewed directly on the CKD site. "

5-iii) Revisions and updating

Revisions and updating. Clearly mention the date and/or version number of the application/intervention (and comparator, if applicable) evaluated, or describe whether the intervention underwent major changes during the evaluation process, or whether the development and/or content was "frozen" during the trial. Describe dynamic components such as news feeds or changing content which may have an impact on the replicability of the intervention (for unexpected events see item 3b).

	1	2	3	4	5	
subitem not at all important	○	○	○	◉	○	essential

Clear selection

Does your paper address subitem 5-iii?

Copy and paste relevant sections from the manuscript (include quotes in quotation marks "like this" to indicate direct quotes from your manuscript), or elaborate on this item by providing additional information not in the ms, or briefly explain why the item is not applicable/relevant for your study

Major updates were not made to the intervention during the study, and therefore are not mentioned

5-iv) Quality assurance methods

Provide information on quality assurance methods to ensure accuracy and quality of information provided [1], if applicable.

	1	2	3	4	5	
subitem not at all important	○	○	○	◉	○	essential

Clear selection

Does your paper address subitem 5-iv?

Copy and paste relevant sections from the manuscript (include quotes in quotation marks "like this" to indicate direct quotes from your manuscript), or elaborate on this item by providing additional information not in the ms, or briefly explain why the item is not applicable/relevant for your study

"Randomisation
Participants were randomized 1:1 to intervention or control. A randomization list was generated in R (v3.3.1),[31] using the package randomizeR (v1.4),[32] which was locked and digitally signed prior to study commencement. A separate randomization list was produced for each of the two study sites. Block randomization approach was used, with random sized blocks of 2-4. After patients had consented to participate in the study, the RN sent the patient ID to the study coordinator, where patients were sequentially allocated against this list."

5-v) Ensure replicability by publishing the source code, and/or providing screenshots/screen-capture video, and/or providing flowcharts of the algorithms used

Ensure replicability by publishing the source code, and/or providing screenshots/screen-capture video, and/or providing flowcharts of the algorithms used. Replicability (i.e., other researchers should in principle be able to replicate the study) is a hallmark of scientific reporting.

	1	2	3	4	5	
subitem not at all important	○	○	○	○	○	essential

Does your paper address subitem 5-v?

Copy and paste relevant sections from the manuscript (include quotes in quotation marks "like this" to indicate direct quotes from your manuscript), or elaborate on this item by providing additional information not in the ms, or briefly explain why the item is not applicable/relevant for your study

Screenshots of the CareKnowDo site provided as a multimedia appendix

5-vi) Digital preservation

Digital preservation: Provide the URL of the application, but as the intervention is likely to change or disappear over the course of the years; also make sure the intervention is archived (Internet Archive, webcitation.org, and/or publishing the source code or screenshots/videos alongside the article). As pages behind login screens cannot be archived, consider creating demo pages which are accessible without login.

	1	2	3	4	5	
subitem not at all important	○	○	○	◉	○	essential

Clear selection

Does your paper address subitem 5-vi?

Copy and paste relevant sections from the manuscript (include quotes in quotation marks "like this" to indicate direct quotes from your manuscript), or elaborate on this item by providing additional information not in the ms, or briefly explain why the item is not applicable/relevant for your study

The intervention is currently commercial in confidence, and not publicly available while still under further development - therefore screenshots have been provided, but full source code has not

5-vii) Access

Access: Describe how participants accessed the application, in what setting/context, if they had to pay (or were paid) or not, whether they had to be a member of specific group. If known, describe how participants obtained "access to the platform and Internet" [1]. To ensure access for editors/reviewers/readers, consider to provide a "backdoor" login account or demo mode for reviewers/readers to explore the application (also important for archiving purposes, see vi).

	1	2	3	4	5	
subitem not at all important	○	○	○	●	○	essential

Clear selection

Does your paper address subitem 5-vii? *

Copy and paste relevant sections from the manuscript (include quotes in quotation marks "like this" to indicate direct quotes from your manuscript), or elaborate on this item by providing additional information not in the ms, or briefly explain why the item is not applicable/relevant for your study

"Due to PV integration, patients in both arms needed to have a PV login before they could proceed. Once randomised, patients in both control and intervention arms were therefore sent an email inviting them to sign-up to PV online. Upon following the link in the email, they were taken to a survey, used for both baseline measurement and program personalisation. Upon completion of the questionnaire, they were taken to the PV homepage (PV group) or taken to the home page for CareKnowDo (CareKnowDo group)."

5-viii) Mode of delivery, features/functionalities/components of the intervention and comparator, and the theoretical framework

Describe mode of delivery, features/functionalities/components of the intervention and comparator, and the theoretical framework [6] used to design them (instructional strategy [1], behaviour change techniques, persuasive features, etc., see e.g., [7, 8] for terminology). This includes an in-depth description of the content (including where it is coming from and who developed it) [1]," whether [and how] it is tailored to individual circumstances and allows users to track their progress and receive feedback" [6]. This also includes a description of communication delivery channels and – if computer-mediated communication is a component – whether communication was synchronous or asynchronous [6]. It also includes information on presentation strategies [1], including page design principles, average amount of text on pages, presence of hyperlinks to other resources, etc. [1].

	1	2	3	4	5	
subitem not at all important	○	○	○	○	●	essential

Clear selection

Does your paper address subitem 5-viii? *

Copy and paste relevant sections from the manuscript (include quotes in quotation marks "like this" to indicate direct quotes from your manuscript), or elaborate on this item by providing additional information not in the ms, or briefly explain why the item is not applicable/relevant for your study

"Patient View

PV is a website that gives patients direct access to their latest test results, and other information such as doctors' letters. It is available to most UK renal patients, but patients must sign up through their renal service.
PV allows patients to remotely log into a secure website that can relay their latest clinic information such as blood test results and doctors' letters. Helping patients to be more involved in their care, by being more aware of their results may improve outcomes[33]. However, uptake is often low, and the site does not offer any behavioral support beyond the results themselves. Additionally, it is important to support patients with resources and education to help them interpret the results that they receive via patient portals such as PV. A study found that 65% of patients incorrectly interpreted the risk presented by their results as presented in a hypothetical scenario, and would likely have taken inappropriate action (calling their doctor immediately, making an appointment within the next 4 weeks, or waiting 3 months for their next appointment)[34].

CareKnowDo
Patients in the CareKnowDo arm had access to a website with three distinct modules. These were:

- Mind Matters: Designed to address low mood
- Lifestyle Matters: Addressing primarily diet, exercise, and how these affect CKD
- Medication Matters: Covering adherence to antihypertensive medication

Each module included interactive activities/tools based on cognitive behavioral therapy and evidence-based behavior change techniques. They also contained psychoeducational content designed to educate patients about CKD and address key unhelpful beliefs impacting on their self-management behavior.

The order in which patients were directed to these modules was determined by how they answered the questionnaire at baseline. These were supported by SMS and emails on each of those topics, directing the patient to the site, and supporting these topics. Patients who scored lower on Necessity and higher on Concerns about medication, based on their scores on the Beliefs about Medicines Questionnaire (see below), were allocated to a 'High risk' profile, and would receive additional nurse calls. Patients could opt out of any of the individual channels, or the program altogether. Each patient was enrolled on the program for six months, after which point email, SMS, calls, and tailoring of the website stopped."

5-ix) Describe use parameters

Describe use parameters (e.g., intended “doses” and optimal timing for use). Clarify what instructions or recommendations were given to the user, e.g., regarding timing, frequency, heaviness of use, if any, or was the intervention used ad libitum.

	1	2	3	4	5	
subitem not at all important	○	○	◉	○	○	essential

Clear selection

Does your paper address subitem 5-ix?

Copy and paste relevant sections from the manuscript (include quotes in quotation marks "like this" to indicate direct quotes from your manuscript), or elaborate on this item by providing additional information not in the ms, or briefly explain why the item is not applicable/relevant for your study

"The order in which patients were directed to these modules was determined by how they answered the questionnaire at baseline. These were supported by SMS and emails on each of those topics, directing the patient to the site, and supporting these topics. Patients who scored lower on Necessity and higher on Concerns about medication, based on their scores on the Beliefs about Medicines Questionnaire (see below), were allocated to a ‘High risk’ profile, and would receive additional nurse calls. Patients could opt out of any of the individual channels, or the program altogether. Each patient was enrolled on the program for six months, after which point email, SMS, calls, and tailoring of the website stopped."

5-x) Clarify the level of human involvement

Clarify the level of human involvement (care providers or health professionals, also technical assistance) in the e-intervention or as co-intervention (detail number and expertise of professionals involved, if any, as well as “type of assistance offered, the timing and frequency of the support, how it is initiated, and the medium by which the assistance is delivered”. It may be necessary to distinguish between the level of human involvement required for the trial, and the level of human involvement required for a routine application outside of a RCT setting (discuss under item 21 – generalizability).

	1	2	3	4	5	
subitem not at all important	○	○	○	◉	○	essential

Clear selection

Does your paper address subitem 5-x?

Copy and paste relevant sections from the manuscript (include quotes in quotation marks "like this" to indicate direct quotes from your manuscript), or elaborate on this item by providing additional information not in the ms, or briefly explain why the item is not applicable/relevant for your study

"Patients who scored lower on Necessity and higher on Concerns about medication, based on their scores on the Beliefs about Medicines Questionnaire (see below), were allocated to a ‘High risk’ profile, and would receive additional nurse calls. Patients could opt out of any of the individual channels, or the program altogether. Each patient was enrolled on the program for six months, after which point email, SMS, calls, and tailoring of the website stopped."

5-xi) Report any prompts/reminders used

Report any prompts/reminders used: Clarify if there were prompts (letters, emails, phone calls, SMS) to use the application, what triggered them, frequency etc. It may be necessary to distinguish between the level of prompts/reminders required for the trial, and the level of prompts/reminders for a routine application outside of a RCT setting (discuss under item 21 – generalizability).

	1	2	3	4	5	
subitem not at all important	○	○	○	◉	○	essential

Clear selection

Does your paper address subitem 5-xi? *

Copy and paste relevant sections from the manuscript (include quotes in quotation marks "like this" to indicate direct quotes from your manuscript), or elaborate on this item by providing additional information not in the ms, or briefly explain why the item is not applicable/relevant for your study

"A reminder email was sent to patients who did not engage with the intervention within the last month."

5-xii) Describe any co-interventions (incl. training/support)

Describe any co-interventions (incl. training/support): Clearly state any interventions that are provided in addition to the targeted eHealth intervention, as ehealth intervention may not be designed as stand-alone intervention. This includes training sessions and support [1]. It may be necessary to distinguish between the level of training required for the trial, and the level of training for a routine application outside of a RCT setting (discuss under item 21 – generalizability.

	1	2	3	4	5	
subitem not at all important	○	○	○	○	○	essential

Does your paper address subitem 5-xii? *

Copy and paste relevant sections from the manuscript (include quotes in quotation marks "like this" to indicate direct quotes from your manuscript), or elaborate on this item by providing additional information not in the ms, or briefly explain why the item is not applicable/relevant for your study

There were no additional interventions beyond the multichannel support described in the manuscript

6a) Completely defined pre-specified primary and secondary outcome measures, including how and when they were assessed

Does your paper address CONSORT subitem 6a? *

Copy and paste relevant sections from the manuscript (include quotes in quotation marks "like this" to indicate direct quotes from your manuscript), or elaborate on this item by providing additional information not in the ms, or briefly explain why the item is not applicable/relevant for your study

"Data collection and outcomes
Demographic details including age, gender, ethnicity, age of leaving full time education were collected via the CareKnowDo website. A wide range of clinical details collected included CKD stage, number of prescribed medications, comorbidities, eGFR (a measure of kidney function), and HbA1c. Additionally, the following psychological measures were captured through online self-assessment:
Beliefs about Medicines Questionnaire (BMQ):34 Based on the Necessity/Concerns Framework of treatment beliefs. This gives an indication of how necessary a person thinks their medicine is (5 items) and how concerned they are about it (5 items).
Brief Illness Perceptions Questionnaire (B-IPQ):35 Based on the Common Sense Model of Illness Representations, this measures patient beliefs about specific aspects of their illness (eg, identity; personal control; treatment control; consequences; emotional impact; timeline; coherence), using single item scales (score range 1-10)
PHQ-9:38 A screening measure of depressive symptoms used in both research, and clinical practice in the UK.
The full list of measures can be found in [appendix A]."

Outcomes
As a feasibility study, no single primary endpoint was selected. Data used to assess feasibility included:
- Uptake: What proportion of patients chose to participate and reasons for declining
- Willingness for patients to be randomized
- Response rates to follow-up questionnaires
- Number of patients enrolled per month
- Means and standard deviations for the outcome measures (e.g. blood pressure) to allow estimation of sample size for a full-powered RCT

Qualitative interviews were conducted with 5 participants after they had completed the six-month trial period. Interviews were conducted by JR by phone, and lasted approximately 30-40 minutes. A semi-structured interview guide was followed, and thematic analysis applied to the findings."

"Follow-up
The initial plan for follow-up was at 12 months, but due to recruitment taking longer than anticipated this was adjusted to 6-months."

6a-i) Online questionnaires: describe if they were validated for online use and apply CHERRIES items to describe how the questionnaires were designed/deployed

If outcomes were obtained through online questionnaires, describe if they were validated for online use and apply CHERRIES items to describe how the questionnaires were designed/deployed [9].

	1	2	3	4	5	
subitem not at all important	○	○	○	◉	○	essential

Clear selection

Does your paper address subitem 6a-i?

Copy and paste relevant sections from manuscript text

"Data collection and outcomes
Demographic details including age, gender, ethnicity, age of leaving full time education were collected via the CareKnowDo website. A wide range of clinical details collected included CKD stage, number of prescribed medications, comorbidities, eGFR (a measure of kidney function), and HbA1c. Additionally, the following psychological measures were captured through online self-assessment:
Beliefs about Medicines Questionnaire (BMQ):34 Based on the Necessity/Concerns Framework of treatment beliefs. This gives an indication of how necessary a person thinks their medicine is (5 items) and how concerned they are about it (5 items).
Brief Illness Perceptions Questionnaire (B-IPQ):35 Based on the Common Sense Model of Illness Representations, this measures patient beliefs about specific aspects of their illness (eg, identity; personal control; treatment control; consequences; emotional impact; timeline; coherence), using single item scales (score range 1-10)
PHQ-9:38 A screening measure of depressive symptoms used in both research, and clinical practice in the UK.
The full list of measures can be found in [appendix A]."

6a-ii) Describe whether and how "use" (including intensity of use/dosage) was defined/measured/monitored

Describe whether and how "use" (including intensity of use/dosage) was defined/measured/monitored (logins, logfile analysis, etc.). Use/adoption metrics are important process outcomes that should be reported in any ehealth trial.

	1	2	3	4	5	
subitem not at all important	○	○	○	◉	○	essential

Clear selection

Does your paper address subitem 6a-ii?

Copy and paste relevant sections from manuscript text

"Response Rates to Follow-up Questionnaires
Once patients were fully enrolled and had completed the baseline questionnaire, 23 patients completed the follow-up questionnaire at 6 months (37.7% of patients starting the study). This constitutes 72.4% of patients who completed enrolment to the intervention.
Due to the high rate of non-completion of the baseline survey (49.2%), clinical differences between the two groups were analyzed. No significant differences in baseline clinical variables were detected between those completing the baseline questionnaire and those not (highest Pearson's r = 0.19, ns, for HbA1c, combined logistic regression model of blood pressure, eGFR, HbA1c, sex and age did not significantly predict baseline survey completion).

Telephone Engagement
Attempts were made to reach patients via phone if they did not complete the baseline survey after a reminder email had been sent. Many patients could not be contacted by phone after 3 attempts. Follow-up revealed that one potential cause could be patients being at work at the time that calls were attempted (9-5 on weekdays), as many patients included in the study were of working age. "

6a-iii) Describe whether, how, and when qualitative feedback from participants was obtained

Describe whether, how, and when qualitative feedback from participants was obtained (e.g., through emails, feedback forms, interviews, focus groups).

	1	2	3	4	5	
subitem not at all important	○	○	○	◉	○	essential

Clear selection

Does your paper address subitem 6a-iii?

Copy and paste relevant sections from manuscript text

"Qualitative Feedback
Qualitative feedback was obtained in the form of free text comments in the 6-month follow up survey available to all participants, and five in-depth qualitative interviews with patients from the intervention arm.
Generally, patients leaving comments found the service helpful, but pointed out some areas for improvement (table 3)."

Full reporting in manuscript

6b) Any changes to trial outcomes after the trial commenced, with reasons

Does your paper address CONSORT subitem 6b? *

Copy and paste relevant sections from the manuscript (include quotes in quotation marks "like this" to indicate direct quotes from your manuscript), or elaborate on this item by providing additional information not in the ms, or briefly explain why the item is not applicable/relevant for your study

Follow-up
The initial plan for follow-up was at 12 months, but due to recruitment taking longer than anticipated this was adjusted to 6-months.

7a) How sample size was determined

NPT: When applicable, details of whether and how the clustering by care provides or centers was addressed

7a-i) Describe whether and how expected attrition was taken into account when calculating the sample size

Describe whether and how expected attrition was taken into account when calculating the sample size.

	1	2	3	4	5	
subitem not at all important	○	○	○	◉	○	essential

Clear selection

Does your paper address subitem 7a-i?

Copy and paste relevant sections from manuscript title (include quotes in quotation marks "like this" to indicate direct quotes from your manuscript), or elaborate on this item by providing additional information not in the ms, or briefly explain why the item is not applicable/relevant for your study

"Sample Size
Sample size was determined pragmatically as a suitable number for the purposes of assessing feasibility."

7b) When applicable, explanation of any interim analyses and stopping guidelines

Does your paper address CONSORT subitem 7b? *

Copy and paste relevant sections from the manuscript (include quotes in quotation marks "like this" to indicate direct quotes from your manuscript), or elaborate on this item by providing additional information not in the ms, or briefly explain why the item is not applicable/relevant for your study

Not applicable - no interim analyses performed

8a) Method used to generate the random allocation sequence
NPT: When applicable, how care providers were allocated to each trial group

Does your paper address CONSORT subitem 8a? *

Copy and paste relevant sections from the manuscript (include quotes in quotation marks "like this" to indicate direct quotes from your manuscript), or elaborate on this item by providing additional information not in the ms, or briefly explain why the item is not applicable/relevant for your study

"Randomisation
Participants were randomized 1:1 to intervention or control. A randomization list was generated in R (v3.3.1),[31] using the package randomizeR (v1.4),[32] which was locked and digitally signed prior to study commencement. A separate randomization list was produced for each of the two study sites. Block randomization approach was used, with random sized blocks of 2-4. After patients had consented to participate in the study, the RN sent the patient ID to the study coordinator, where patients were sequentially allocated against this list."

8b) Type of randomisation; details of any restriction (such as blocking and block size)

Does your paper address CONSORT subitem 8b? *

Copy and paste relevant sections from the manuscript (include quotes in quotation marks "like this" to indicate direct quotes from your manuscript), or elaborate on this item by providing additional information not in the ms, or briefly explain why the item is not applicable/relevant for your study

"Randomisation
Participants were randomized 1:1 to intervention or control. A randomization list was generated in R (v3.3.1),[31] using the package randomizeR (v1.4),[32] which was locked and digitally signed prior to study commencement. A separate randomization list was produced for each of the two study sites. Block randomization approach was used, with random sized blocks of 2-4. After patients had consented to participate in the study, the RN sent the patient ID to the study coordinator, where patients were sequentially allocated against this list."

9) Mechanism used to implement the random allocation sequence (such as sequentially numbered containers), describing any steps taken to conceal the sequence until interventions were assigned

Does your paper address CONSORT subitem 9? *

Copy and paste relevant sections from the manuscript (include quotes in quotation marks "like this" to indicate direct quotes from your manuscript), or elaborate on this item by providing additional information not in the ms, or briefly explain why the item is not applicable/relevant for your study

"Randomisation
Participants were randomized 1:1 to intervention or control. A randomization list was generated in R (v3.3.1),[31] using the package randomizeR (v1.4),[32] which was locked and digitally signed prior to study commencement. A separate randomization list was produced for each of the two study sites. Block randomization approach was used, with random sized blocks of 2-4. After patients had consented to participate in the study, the RN sent the patient ID to the study coordinator, where patients were sequentially allocated against this list."

10) Who generated the random allocation sequence, who enrolled participants, and who assigned participants to interventions

Does your paper address CONSORT subitem 10? *

Copy and paste relevant sections from the manuscript (include quotes in quotation marks "like this" to indicate direct quotes from your manuscript), or elaborate on this item by providing additional information not in the ms, or briefly explain why the item is not applicable/relevant for your study

"Randomisation
Participants were randomized 1:1 to intervention or control. A randomization list was generated in R (v3.3.1),[31] using the package randomizeR (v1.4),[32] which was locked and digitally signed prior to study commencement. A separate randomization list was produced for each of the two study sites. Block randomization approach was used, with random sized blocks of 2-4. After patients had consented to participate in the study, the RN sent the patient ID to the study coordinator, where patients were sequentially allocated against this list."

11a) If done, who was blinded after assignment to interventions (for example, participants, care providers, those assessing outcomes) and how

NPT: Whether or not administering co-interventions were blinded to group assignment

11a-i) Specify who was blinded, and who wasn't

Specify who was blinded, and who wasn't. Usually, in web-based trials it is not possible to blind the participants [1, 3] (this should be clearly acknowledged), but it may be possible to blind outcome assessors, those doing data analysis or those administering co-interventions (if any).

	1	2	3	4	5	
subitem not at all important	○	○	○	◉	○	essential

Clear selection

Does your paper address subitem 11a-i? *

Copy and paste relevant sections from the manuscript (include quotes in quotation marks "like this" to indicate direct quotes from your manuscript), or elaborate on this item by providing additional information not in the ms, or briefly explain why the item is not applicable/relevant for your study

Blinding was not used per se, but participants were not informed about the in-depth details of CareKnowDo as an intervention unless they were randomized to receive it,

"The patient information sheet included information on the process of randomization into one of the two groups, and a broad idea of what the supportive intervention and control group would entail. Neither group were given in-depth details about the contents of CareKnowDo until after randomisation."

11a-ii) Discuss e.g., whether participants knew which intervention was the “intervention of interest” and which one was the “comparator”

Informed consent procedures (4a-ii) can create biases and certain expectations - discuss e.g., whether participants knew which intervention was the “intervention of interest” and which one was the “comparator”.

	1	2	3	4	5	
subitem not at all important	○	○	○	●	○	essential

Clear selection

Does your paper address subitem 11a-ii?

Copy and paste relevant sections from the manuscript (include quotes in quotation marks "like this" to indicate direct quotes from your manuscript), or elaborate on this item by providing additional information not in the ms, or briefly explain why the item is not applicable/relevant for your study

It is clear in the paper that Patient View is something patients already have access to, and that CareKnowDo is a new intervention

11b) If relevant, description of the similarity of interventions

(this item is usually not relevant for ehealth trials as it refers to similarity of a placebo or sham intervention to a active medication/intervention)

Does your paper address CONSORT subitem 11b? *

Copy and paste relevant sections from the manuscript (include quotes in quotation marks "like this" to indicate direct quotes from your manuscript), or elaborate on this item by providing additional information not in the ms, or briefly explain why the item is not applicable/relevant for your study

"PV allows people being treated for kidney disease by the NHS to view the results of their clinical tests, such as blood and urine tests, as well as recent letters from their nephrologist. Part of the rationale for this is supporting people living with CKD to make decisions, and to take actions, that will reduce the likelihood of progression to ESKD. However, PV provides little information about what to do with this information, or how translate it into behaviour change, such as reducing salt intake, adhering to medicines, or increasing physical activity. CareKnowDo is a multichannel support programme, developed by a team of doctoral level psychologists at Atlantis Health, in collaboration with the Renal Association, that aims to address this gap. It does so by providing people living with CKD with tailored web, SMS, email, and nurse phone support based around these types of self-management behaviour. It also features integration with PV, so that results can be viewed directly on the CKD site.
As CareKnowDo (with PV integration) was a novel intervention, the core aim of the study was to establish the feasibility of rolling this out in NHS kidney units, establish uptake, and examine facilitators and barriers to inform future intervention and trial design[28]. Trial design and reporting were guided by the Pilot and Feasibility extension of the 2010 CONSORT guidelines[29]."

12a) Statistical methods used to compare groups for primary and secondary outcomes

NPT: When applicable, details of whether and how the clustering by care providers or centers was addressed

Does your paper address CONSORT subitem 12a? *

Copy and paste relevant sections from the manuscript (include quotes in quotation marks "like this" to indicate direct quotes from your manuscript), or elaborate on this item by providing additional information not in the ms, or briefly explain why the item is not applicable/relevant for your study

"Analysis Approach
Due to the feasibility nature of the study, statistical analysis was predominantly descriptive. Indications of efficacy were investigated using correlation and regression conducted in R. Exploratory correlational analysis controlled for multiple comparisons using Bonferroni correction."

12a-i) Imputation techniques to deal with attrition / missing values

Imputation techniques to deal with attrition / missing values: Not all participants will use the intervention/comparator as intended and attrition is typically high in ehealth trials. Specify how participants who did not use the application or dropped out from the trial were treated in the statistical analysis (a complete case analysis is strongly discouraged, and simple imputation techniques such as LOCF may also be problematic [4]).

	1	2	3	4	5	
subitem not at all important	○	○	○	○	◉	essential

Clear selection

Does your paper address subitem 12a-i? *

Copy and paste relevant sections from the manuscript (include quotes in quotation marks "like this" to indicate direct quotes from your manuscript), or elaborate on this item by providing additional information not in the ms, or briefly explain why the item is not applicable/relevant for your study

"Analysis Approach
Due to the feasibility nature of the study, statistical analysis was predominantly descriptive. Indications of efficacy were investigated using correlation and regression conducted in R. Exploratory correlational analysis controlled for multiple comparisons using Bonferroni correction. An Intention to Treat (ITT) approach was taken where dropout occurred, and missing data were not imputed."

12b) Methods for additional analyses, such as subgroup analyses and adjusted analyses

Does your paper address CONSORT subitem 12b? *

Copy and paste relevant sections from the manuscript (include quotes in quotation marks "like this" to indicate direct quotes from your manuscript), or elaborate on this item by providing additional information not in the ms, or briefly explain why the item is not applicable/relevant for your study

2Analysis Approach
Due to the feasibility nature of the study, statistical analysis was predominantly descriptive. Indications of efficacy were investigated using correlation and regression conducted in R. Exploratory correlational analysis controlled for multiple comparisons using Bonferroni correction. An Intention to Treat (ITT) approach was taken where dropout occurred, and missing data were not imputed..
Qualitative interviews were directly audio coded using the principles of thematic analysis[35]."

X26) REB/IRB Approval and Ethical Considerations [recommended as subheading under "Methods"] (not a CONSORT item)

X26-i) Comment on ethics committee approval

	1	2	3	4	5	
subitem not at all important	○	○	○	◉	○	essential

Clear selection

Does your paper address subitem X26-i?

Copy and paste relevant sections from the manuscript (include quotes in quotation marks "like this" to indicate direct quotes from your manuscript), or elaborate on this item by providing additional information not in the ms, or briefly explain why the item is not applicable/relevant for your study

"Ethics & Informed Consent
Approval for the study was given by the London Dulwich Research Ethics Committee on 14/12/2015, reference 15/LO/1700. Written consent was required for all patients prior to their participant in the study. Participants were free to withdraw at any time."

x26-ii) Outline informed consent procedures

Outline informed consent procedures e.g., if consent was obtained offline or online (how? Checkbox, etc.?), and what information was provided (see 4a-ii). See [6] for some items to be included in informed consent documents.

	1	2	3	4	5	
subitem not at all important	○	◉	○	○	○	essential

Clear selection

Does your paper address subitem X26-ii?

Copy and paste relevant sections from the manuscript (include quotes in quotation marks "like this" to indicate direct quotes from your manuscript), or elaborate on this item by providing additional information not in the ms, or briefly explain why the item is not applicable/relevant for your study

"Ethics & Informed Consent
Approval for the study was given by the London Dulwich Research Ethics Committee on 14/12/2015, reference 15/LO/1700. Written consent was required for all patients prior to their participant in the study. Participants were free to withdraw at any time."

X26-iii) Safety and security procedures

Safety and security procedures, incl. privacy considerations, and any steps taken to reduce the likelihood or detection of harm (e.g., education and training, availability of a hotline)

	1	2	3	4	5	
subitem not at all important	○	○	○	◉	○	essential

Clear selection

Does your paper address subitem X26-iii?

Copy and paste relevant sections from the manuscript (include quotes in quotation marks "like this" to indicate direct quotes from your manuscript), or elaborate on this item by providing additional information not in the ms, or briefly explain why the item is not applicable/relevant for your study

"Patients were also provided with an inbound nurse line for queries or concerns."

RESULTS

13a) For each group, the numbers of participants who were randomly assigned, received intended treatment, and were analysed for the primary outcome

NPT: The number of care providers or centers performing the intervention in each group and the number of patients treated by each care provider in each center

Does your paper address CONSORT subitem 13a? *

Copy and paste relevant sections from the manuscript (include quotes in quotation marks "like this" to indicate direct quotes from your manuscript), or elaborate on this item by providing additional information not in the ms, or briefly explain why the item is not applicable/relevant for your study

Yes, see CONSORT flow diagrams and tables of participant characteristics

13b) For each group, losses and exclusions after randomisation, together with reasons

Does your paper address CONSORT subitem 13b? (NOTE: Preferably, this is shown in a CONSORT flow diagram) *

Copy and paste relevant sections from the manuscript (include quotes in quotation marks "like this" to indicate direct quotes from your manuscript), or elaborate on this item by providing additional information not in the ms, or briefly explain why the item is not applicable/relevant for your study

Yes, see CONSORT flow diagram

13b-i) Attrition diagram

Strongly recommended: An attrition diagram (e.g., proportion of participants still logging in or using the intervention/comparator in each group plotted over time, similar to a survival curve) or other figures or tables demonstrating usage/dose/engagement.

	1	2	3	4	5	
subitem not at all important	○	○	○	◉	○	essential

Clear selection

Does your paper address subitem 13b-i?

Copy and paste relevant sections from the manuscript or cite the figure number if applicable (include quotes in quotation marks "like this" to indicate direct quotes from your manuscript), or elaborate on this item by providing additional information not in the ms, or briefly explain why the item is not applicable/relevant for your study

Yes, see attrition diagram

14a) Dates defining the periods of recruitment and follow-up

Does your paper address CONSORT subitem 14a? *

Copy and paste relevant sections from the manuscript (include quotes in quotation marks "like this" to indicate direct quotes from your manuscript), or elaborate on this item by providing additional information not in the ms, or briefly explain why the item is not applicable/relevant for your study

Follow-up

"The initial plan for follow-up was at 12 months, but due to recruitment taking longer than anticipated this was adjusted to 6-months."

14a-i) Indicate if critical “secular events” fell into the study period

Indicate if critical “secular events” fell into the study period, e.g., significant changes in Internet resources available or “changes in computer hardware or Internet delivery resources”

	1	2	3	4	5	
subitem not at all important	○	◉	○	○	○	essential

Clear selection

Does your paper address subitem 14a-i?

Copy and paste relevant sections from the manuscript (include quotes in quotation marks "like this" to indicate direct quotes from your manuscript), or elaborate on this item by providing additional information not in the ms, or briefly explain why the item is not applicable/relevant for your study

No secular events fell into the study period, and so are not described

14b) Why the trial ended or was stopped (early)

Does your paper address CONSORT subitem 14b? *

Copy and paste relevant sections from the manuscript (include quotes in quotation marks "like this" to indicate direct quotes from your manuscript), or elaborate on this item by providing additional information not in the ms, or briefly explain why the item is not applicable/relevant for your study

The trial was not ended early, but the follow-up period was shortened due to recruitment taking longer than anticipated. Reasons are provided.

"Initially, participants in each arm were to be stratified into 'prevalent' and 'incident' patients, with a recruitment target of 30 for each group, (to capture potential differences between these groups. It was purported that the intervention would be particularly valuable to newer patients. Patients were considered 'prevalent; if they:

- Had been invited to attend three nephology outpatient clinics in the last 12 months
- Attended nephrology outpatient clinics at least twice previously

However, due to a lack of patients being recruited in the incident stratum, this stratification requirement was dropped, and the final sample consisted predominantly of prevalent patients (n = 54 out of 61)."

"Follow-up
The initial plan for follow-up was at 12 months, but due to recruitment taking longer than anticipated this was adjusted to 6-months."

15) A table showing baseline demographic and clinical characteristics for each group

NPT: When applicable, a description of care providers (case volume, qualification, expertise, etc.) and centers (volume) in each group

Does your paper address CONSORT subitem 15? *

Copy and paste relevant sections from the manuscript (include quotes in quotation marks "like this" to indicate direct quotes from your manuscript), or elaborate on this item by providing additional information not in the ms, or briefly explain why the item is not applicable/relevant for your study

Yes, this is provided in Table 1

15-i) Report demographics associated with digital divide issues

In ehealth trials it is particularly important to report demographics associated with digital divide issues, such as age, education, gender, social-economic status, computer/Internet/ehealth literacy of the participants, if known.

	1	2	3	4	5	
subitem not at all important	○	○	○	◉	○	essential

Clear selection

Does your paper address subitem 15-i? *

Copy and paste relevant sections from the manuscript (include quotes in quotation marks "like this" to indicate direct quotes from your manuscript), or elaborate on this item by providing additional information not in the ms, or briefly explain why the item is not applicable/relevant for your study

Yes, these are covered in Table 1

16) For each group, number of participants (denominator) included in each analysis and whether the analysis was by original assigned groups

16-i) Report multiple "denominators" and provide definitions

Report multiple "denominators" and provide definitions: Report N's (and effect sizes) "across a range of study participation [and use] thresholds" [1], e.g., N exposed, N consented, N used more than x times, N used more than y weeks, N participants "used" the intervention/comparator at specific pre-defined time points of interest (in absolute and relative numbers per group). Always clearly define "use" of the intervention.

	1	2	3	4	5	
subitem not at all important	○	○	○	◉	○	essential

Clear selection

Does your paper address subitem 16-i? *

Copy and paste relevant sections from the manuscript (include quotes in quotation marks "like this" to indicate direct quotes from your manuscript), or elaborate on this item by providing additional information not in the ms, or briefly explain why the item is not applicable/relevant for your study

Yes, absolute and relative values are given, including the denominators for each set of statistics

16-ii) Primary analysis should be intent-to-treat

Primary analysis should be intent-to-treat, secondary analyses could include comparing only "users", with the appropriate caveats that this is no longer a randomized sample (see 18-i).

	1	2	3	4	5	
subitem not at all important	○	○	○	◉	○	essential

Clear selection

Does your paper address subitem 16-ii?

Copy and paste relevant sections from the manuscript (include quotes in quotation marks "like this" to indicate direct quotes from your manuscript), or elaborate on this item by providing additional information not in the ms, or briefly explain why the item is not applicable/relevant for your study

Yes, analysis is on an ITT basis

"Analysis Approach
Due to the feasibility nature of the study, statistical analysis was predominantly descriptive. Indications of efficacy were investigated using correlation and regression conducted in R. Exploratory correlational analysis controlled for multiple comparisons using Bonferroni correction. An Intention to Treat (ITT) approach was taken where dropout occurred, and missing data were not imputed."

17a) For each primary and secondary outcome, results for each group, and the estimated effect size and its precision (such as 95% confidence interval)

Does your paper address CONSORT subitem 17a? *

Copy and paste relevant sections from the manuscript (include quotes in quotation marks "like this" to indicate direct quotes from your manuscript), or elaborate on this item by providing additional information not in the ms, or briefly explain why the item is not applicable/relevant for your study

Yes, where relevant effect sizes and standard deviations are used

17a-i) Presentation of process outcomes such as metrics of use and intensity of use

In addition to primary/secondary (clinical) outcomes, the presentation of process outcomes such as metrics of use and intensity of use (dose, exposure) and their operational definitions is critical. This does not only refer to metrics of attrition (13-b) (often a binary variable), but also to more continuous exposure metrics such as “average session length”. These must be accompanied by a technical description how a metric like a “session” is defined (e.g., timeout after idle time) [1] (report under item 6a).

	1	2	3	4	5	
subitem not at all important	○	○	○	◉	○	essential

Clear selection

Does your paper address subitem 17a-i?

Copy and paste relevant sections from the manuscript (include quotes in quotation marks "like this" to indicate direct quotes from your manuscript), or elaborate on this item by providing additional information not in the ms, or briefly explain why the item is not applicable/relevant for your study

Engagement throughout study flow is described, and qualitative insights into use are explored in the manuscript. Session use is not described.

17b) For binary outcomes, presentation of both absolute and relative effect sizes is recommended

Does your paper address CONSORT subitem 17b? *

Copy and paste relevant sections from the manuscript (include quotes in quotation marks "like this" to indicate direct quotes from your manuscript), or elaborate on this item by providing additional information not in the ms, or briefly explain why the item is not applicable/relevant for your study

All changes in e.g. engaged/no longer engaged are presented as both absolute and relative values

18) Results of any other analyses performed, including subgroup analyses and adjusted analyses, distinguishing pre-specified from exploratory

Does your paper address CONSORT subitem 18? *

Copy and paste relevant sections from the manuscript (include quotes in quotation marks "like this" to indicate direct quotes from your manuscript), or elaborate on this item by providing additional information not in the ms, or briefly explain why the item is not applicable/relevant for your study

"Exploratory Outcomes Analysis

Part of the rationale of the program was the use of illness perceptions and beliefs about medicines to tailor the program, with a view to reducing blood pressure at follow-up. Perceptions of how much treatment can improve one's condition were negatively correlated with systolic blood pressure at baseline; people who believed treatment could help had lower baseline blood pressure (Pearson's $r = -.52$, $P = .005$), but this relationship was not present at follow-up. Conversely, perceived understanding of illness at baseline is negatively correlated with systolic blood pressure at follow-up (Pearson's $r = -0.66$, $P < .001$). Someone who feels they understand their illness better has lower blood pressure 6 months later. The relationship between Understanding and BP is not present at baseline ($r = -0.31$, $P = .12$). Beliefs about medicines at baseline were associated with blood pressure at baseline, but not at follow-up. This was true for both concerns about medicines ($r = .58$, $P = .001$) and perceived necessity of medicines ($r = .42$, $P = .03$). Patients who had higher concerns about medicines, and those who perceived medication to be more necessary, had higher blood pressure at baseline."

18-i) Subgroup analysis of comparing only users

A subgroup analysis of comparing only users is not uncommon in ehealth trials, but if done, it must be stressed that this is a self-selected sample and no longer an unbiased sample from a randomized trial (see 16-iii).

	1	2	3	4	5	
subitem not at all important	○	●	○	○	○	essential

Clear selection

Does your paper address subitem 18-i?

Copy and paste relevant sections from the manuscript (include quotes in quotation marks "like this" to indicate direct quotes from your manuscript), or elaborate on this item by providing additional information not in the ms, or briefly explain why the item is not applicable/relevant for your study

This type of subgroup analysis was not performed

19) All important harms or unintended effects in each group
(for specific guidance see CONSORT for harms)

Does your paper address CONSORT subitem 19? *

Copy and paste relevant sections from the manuscript (include quotes in quotation marks "like this" to indicate direct quotes from your manuscript), or elaborate on this item by providing additional information not in the ms, or briefly explain why the item is not applicable/relevant for your study

Qualitative interviews gave an opportunity to identify unintended harms - none were found

19-i) Include privacy breaches, technical problems

Include privacy breaches, technical problems. This does not only include physical “harm” to participants, but also incidents such as perceived or real privacy breaches [1], technical problems, and other unexpected/unintended incidents. “Unintended effects” also includes unintended positive effects [2].

	1	2	3	4	5	
subitem not at all important	○	○	○	●	○	essential

Clear selection

Does your paper address subitem 19-i?

Copy and paste relevant sections from the manuscript (include quotes in quotation marks "like this" to indicate direct quotes from your manuscript), or elaborate on this item by providing additional information not in the ms, or briefly explain why the item is not applicable/relevant for your study

Minor technical issues with patients accessing the site are referenced

"Figure 2 shows patient flow after randomization. In each arm, roughly half of participants did not complete enrollment onto the intervention after being randomized. Follow-up RN calls revealed that the most common reasons for this were:

- Not receiving a PV login before attempting to enroll
- Losing invitation emails in spam folders
- Not checking emails
- Technical difficulties logging in"

19-ii) Include qualitative feedback from participants or observations from staff/researchers

Include qualitative feedback from participants or observations from staff/researchers, if available, on strengths and shortcomings of the application, especially if they point to unintended/unexpected effects or uses. This includes (if available) reasons for why people did or did not use the application as intended by the developers.

	1	2	3	4	5	
subitem not at all important	○	○	○	◉	○	essential

Clear selection

Does your paper address subitem 19-ii?

Copy and paste relevant sections from the manuscript (include quotes in quotation marks "like this" to indicate direct quotes from your manuscript), or elaborate on this item by providing additional information not in the ms, or briefly explain why the item is not applicable/relevant for your study

"Reasons for low uptake
Research nurses at each recruiting site captured the reasons for low incident patient recruitment:
1. There were fewer incident patients coming through their clinics than prevalent patients
2. Incident patients frequently did not meet the other inclusion criteria for the study. For example, they were often not on anti-hypertensive medication
3. Some incident patients, or their carers, did not fully acknowledge that they had a chronic kidney condition"

DISCUSSION

22) Interpretation consistent with results, balancing benefits and harms, and considering other relevant evidence

NPT: In addition, take into account the choice of the comparator, lack of or partial blinding, and unequal expertise of care providers or centers in each group

22-i) Restate study questions and summarize the answers suggested by the data, starting with primary outcomes and process outcomes (use)

Restate study questions and summarize the answers suggested by the data, starting with primary outcomes and process outcomes (use).

	1	2	3	4	5	
subitem not at all important	○	○	○	◉	○	essential

Clear selection

Does your paper address subitem 22-i? *

Copy and paste relevant sections from the manuscript (include quotes in quotation marks "like this" to indicate direct quotes from your manuscript), or elaborate on this item by providing additional information not in the ms, or briefly explain why the item is not applicable/relevant for your study

"This feasibility study found that a remote intervention to support self-management of CKD was feasible, and perceived as helpful by participants. A number of areas for improvement were identified."

22-ii) Highlight unanswered new questions, suggest future research

Highlight unanswered new questions, suggest future research.

	1	2	3	4	5	
subitem not at all important	○	○	○	○	○	essential

Does your paper address subitem 22-ii?

Copy and paste relevant sections from the manuscript (include quotes in quotation marks "like this" to indicate direct quotes from your manuscript), or elaborate on this item by providing additional information not in the ms, or briefly explain why the item is not applicable/relevant for your study

"Implications for Future Research and Practice

This study did not manage to create sufficient patient engagement at the start of the program. Gaining this early engagement means addressing perceptions of illness coherence from the introduction of the service to the patient, and even earlier. Such programs designed to prevent future harm, rather than address an immediate set of symptoms or concerns, must be introduced in a way that conveys their purpose, and that this aligns with the patients perceptions of what their illness is – either by adjusting how the program is presented, or by ensuring the patient receives appropriate illness education, including fostering a stronger belief in the need for treatment at this relatively early stage.
Streamlining the enrolment process for online programs is key, even in the more involved context of research. Ensuring that when a patient signs up for support does not require a further period of waiting to first log on is key; engagement falls off quickly. Where possible, patients should be directed to the service by a person at the point at which they have everything they need to proceed.
In future it may be that an intervention should be available to all people diagnosed with CKD, regardless of use of antihypertensive medication, and 'switch on' medication specific components when they become relevant."

20) Trial limitations, addressing sources of potential bias, imprecision, and, if relevant, multiplicity of analyses

20-i) Typical limitations in ehealth trials

Typical limitations in ehealth trials: Participants in ehealth trials are rarely blinded. Ehealth trials often look at a multiplicity of outcomes, increasing risk for a Type I error. Discuss biases due to non-use of the intervention/usability issues, biases through informed consent procedures, unexpected events.

	1	2	3	4	5	
subitem not at all important	○	○	○	●	○	essential

Clear selection

Does your paper address subitem 20-i? *

Copy and paste relevant sections from the manuscript (include quotes in quotation marks "like this" to indicate direct quotes from your manuscript), or elaborate on this item by providing additional information not in the ms, or briefly explain why the item is not applicable/relevant for your study

"This study did not manage to create sufficient patient engagement at the start of the program. Gaining this early engagement means addressing perceptions of illness coherence from the introduction of the service to the patient, and even earlier. Such programs designed to prevent future harm, rather than address an immediate set of symptoms or concerns, must be introduced in a way that conveys their purpose, and that this aligns with the patients perceptions of what their illness is – either by adjusting how the program is presented, or by ensuring the patient receives appropriate illness education, including fostering a stronger belief in the need for treatment at this relatively early stage. "

21) Generalisability (external validity, applicability) of the trial findings

NPT: External validity of the trial findings according to the intervention, comparators, patients, and care providers or centers involved in the trial

21-i) Generalizability to other populations

Generalizability to other populations: In particular, discuss generalizability to a general Internet population, outside of a RCT setting, and general patient population, including applicability of the study results for other organizations

	1	2	3	4	5	
subitem not at all important	○	○	○	○	○	essential

Does your paper address subitem 21-i?

Copy and paste relevant sections from the manuscript (include quotes in quotation marks "like this" to indicate direct quotes from your manuscript), or elaborate on this item by providing additional information not in the ms, or briefly explain why the item is not applicable/relevant for your study

"The content of the program was aimed at both prevalent and incident patients. It may be that the content was not advanced enough for people who had been living with the condition for longer. A future iteration of the program should tailor to whether patients are incident or prevalent, or perceived knowledge about the condition. Additionally, if the program is to address patients not on antihypertensive medication, this should also be included in the tailoring process.
It appears that support from a service such as CareKnowDo was perceived as being most useful to newly diagnosed patients who had the most questions, and had not had years to build up their own experiences of how to live with the disease. However, these are the patients who were most difficult to reach in this study, due in part of a lack of acceptance about their condition. It may be that patients who perceive their current need to be low overestimate how well controlled their CKD is, as ongoing clinic attendance may reflect a high risk of progression. This may reflect unhelpful thoughts or behaviors."

21-ii) Discuss if there were elements in the RCT that would be different in a routine application setting

Discuss if there were elements in the RCT that would be different in a routine application setting (e.g., prompts/reminders, more human involvement, training sessions or other co-interventions) and what impact the omission of these elements could have on use, adoption, or outcomes if the intervention is applied outside of a RCT setting.

	1	2	3	4	5	
subitem not at all important	○	○	○	◉	○	essential

Clear selection

Does your paper address subitem 21-ii?

Copy and paste relevant sections from the manuscript (include quotes in quotation marks "like this" to indicate direct quotes from your manuscript), or elaborate on this item by providing additional information not in the ms, or briefly explain why the item is not applicable/relevant for your study

There would be minimal differences in a routine setting

OTHER INFORMATION

23) Registration number and name of trial registry

Does your paper address CONSORT subitem 23? *

Copy and paste relevant sections from the manuscript (include quotes in quotation marks "like this" to indicate direct quotes from your manuscript), or elaborate on this item by providing additional information not in the ms, or briefly explain why the item is not applicable/relevant for your study

The study was pre-registered with the UK's Health Research Authority, IRAS ID 184206

24) Where the full trial protocol can be accessed, if available

Does your paper address CONSORT subitem 24? *

Cite a Multimedia Appendix, other reference, or copy and paste relevant sections from the manuscript (include quotes in quotation marks "like this" to indicate direct quotes from your manuscript), or elaborate on this item by providing additional information not in the ms, or briefly explain why the item is not applicable/relevant for your study

The full trial protocol is not available online, but can be made available upon request

25) Sources of funding and other support (such as supply of drugs), role of funders

Does your paper address CONSORT subitem 25? *

Copy and paste relevant sections from the manuscript (include quotes in quotation marks "like this" to indicate direct quotes from your manuscript), or elaborate on this item by providing additional information not in the ms, or briefly explain why the item is not applicable/relevant for your study

"This work was funded by the National Institute for Health Research (NIHR) Small Business Research Initiative (SBRI) via Devices for Dignity (D4D), and by Atlantis Healthcare. D4D monitored the study through reporting and steering meetings. Atlantis Healthcare was responsible for the study design, and analysis and interpretation of data. Patient recruitment and data collection were conducted by University Hospitals Bristol NHS Foundation Trust and Gloucestershire Hospitals NHS Foundation Trust."

X27) Conflicts of Interest (not a CONSORT item)

X27-i) State the relation of the study team towards the system being evaluated

In addition to the usual declaration of interests (financial or otherwise), also state the relation of the study team towards the system being evaluated, i.e., state if the authors/evaluators are distinct from or identical with the developers/sponsors of the intervention.

	1	2	3	4	5	
subitem not at all important	○	○	○	◉	○	essential

Clear selection

Does your paper address subitem X27-i?

Copy and paste relevant sections from the manuscript (include quotes in quotation marks "like this" to indicate direct quotes from your manuscript), or elaborate on this item by providing additional information not in the ms, or briefly explain why the item is not applicable/relevant for your study

" Conflict of Interest
JR is an employee of Atlantis Healthcare. JW is a consultant to Atlantis Healthcare. FC, BH, and UU received funding from SBRI and Atlantis Healthcare to work on this project."

The study team collaborated on the development of the intervention. However the research nurses and intervention nurses with direct patient contact were not part of intervention development.

About the CONSORT EHEALTH checklist

As a result of using this checklist, did you make changes in your manuscript? *

- ○ yes, major changes
- ◉ yes, minor changes
- ○ no

What were the most important changes you made as a result of using this checklist?

Additional individual lines to add specific clarification of an existing statements

How much time did you spend on going through the checklist INCLUDING making changes in your manuscript *

1.5 hours in total - the majority of time was spend finding and selecting appropriate quotes, followed by making minor amends

As a result of using this checklist, do you think your manuscript has improved? *

- (x) yes
- ( ) no
- ( ) Other:

Would you like to become involved in the CONSORT EHEALTH group?

This would involve for example becoming involved in participating in a workshop and writing an "Explanation and Elaboration" document

- ( ) yes
- (x) no
- ( ) Other:

Clear selection

Any other comments or questions on CONSORT EHEALTH

An estimate at the start of the form, including whether it can be saved partway through, would make the process considerably more user friendly and practical

STOP - Save this form as PDF before you click submit

To generate a record that you filled in this form, we recommend to generate a PDF of this page (on a Mac, simply select "print" and then select "print as PDF") before you submit it.

When you submit your (revised) paper to JMIR, please upload the PDF as supplementary file.

Don't worry if some text in the textboxes is cut off, as we still have the complete information in our database. Thank you!

Final step: Click submit !

Click submit so we have your answers in our database!

Submit Clear form

Never submit passwords through Google Forms.

This content is neither created nor endorsed by Google. Report Abuse - Terms of Service - Privacy Policy

Google Forms
